# Examining the interactions of Galahad™ compound with viruses to develop a novel inactivated influenza A virus vaccine

**DOI:** 10.1016/j.heliyon.2022.e09887

**Published:** 2022-07-06

**Authors:** Dale L. Barnard, David M. Belnap, Parastoo Azadi, Christian Heiss, D. Scott Snyder, Susan C. Bock, Thomas W. Konowalchuk

**Affiliations:** a ADVS Department, Institute for Antiviral Research, Utah State University, Logan, Utah 84041, United States; b School of Biological Sciences and Department of Biochemistry, University of Utah, Salt Lake City, Utah 84112, United States; c Complex Carbohydrate Research Center, 315 Riverbend Road Athens, GA 30602-4712, United States; d Snyder SutroVax Inc., Foster City, CA, United States; e Biomedical Engineering Department, University of Utah, Salt Lake City, Utah 84112, United States; f Galaxy Force Technologies, LLC 1070 NE 7th Drive, Newport, OR 97365-2518, United States

**Keywords:** Vaccine, Polysaccharide, Catechin, Influenza, H5N1, SARS-CoV-2 (COVID-19)

## Abstract

Galahad™ is a proanthocyanidin complexed with polysaccharides that inactivates viruses and indicates potential for an innovative approach to making protective vaccines. The polysaccharide portion of Galahad™ consists mainly of arabinan and arabinogalactan. In a seven-day toxicity study in rats, it was not toxic even when tested undiluted. Galahad™ inactivated a wide range of DNA and RNA viruses including adenoviruses, corona viruses such as SARS-CoV-2, and influenza viruses. Electron microscopy studies showed that exposure to Galahad™ caused extensive clumping of virions followed by lack of detection of virions after longer periods of exposure. Based on the viral inactivation data, the hypotheses tested is that Galahad™ inactivation of virus can be used to formulate a protective inactivated virus vaccine. To evaluate this hypothesis, infectious influenza A virus (H5N1, Duck/MN/1525/81) with a titer of 10^5.7^ CCID_50_/0.1 ml was exposed for 10 min to Galahad™. This treatment caused the infectious virus titer to be reduced to below detectable limits. The Galahad™ -inactivated influenza preparation without adjuvant or preservative was given to BALB/c mice using a variety of routes of administration and dosing regimens. The most protective route of administration and dosing regimen was when mice were given the vaccine twice intranasally, the second dose coming 14 days after the primary vaccine dose. All the mice receiving this vaccine regimen survived the virus challenge while only 20% of the mice receiving placebo survived. This suggests that a Galahad™-inactivated influenza virus vaccine can elicit a protective immune response even without the use of an adjuvant. This technology should be investigated further for its potential to make effective human vaccines.

## Highlights

• Discovery of novel virus-inactivating agent: polysaccharide/catechin (Galahad™).
• Non-toxic: inactivates/lowers titers in 25 viruses, including Avian H5N1, COVID-19.
• Agent used to make whole virus vaccine; tested in 400 animal mouse model.
• Galahad™-inactivated vaccine 100% successful intranasally vs. lethal H5N1 challenge.
• Platform offers possible benefits—in time, cost, & mass distribution.

## 1 Introduction

Many reports have described health benefits that can be derived from consumption of grape byproducts and extracts prepared from grape seed [1, 2, 3, 4, 5]. At least some of these effects have been attributed to flavonoid constituents of a hot water extract of grape seed [5], and virucidal properties have also been detected [6]. One such compound derived from hot water extract of grape seed is a high molecular weight polysaccharide complexed with a catechin. In a previous study, a catechin purified from a green tea extract was shown to be a virucidal agent [7]. In that study, the catechin was used to inactivate influenza A virus and the product from that inactivation (whole virus vaccine) was shown to be an effective vaccine in mice without using an adjuvant [7]. Such inactivated whole virus vaccines seem to induce stronger immune responses in immunologically naive individuals than other types of vaccines leading to protection against a virus infection [8].

To create inactivated whole virus vaccines, the most commonly used inactivating agents are formalin and β-propiolactone. However, formalin modifies vaccine antigens to such a great extent that immunogenicity of the inactivated virus decreases. In some cases, that modification by formalin, when used to inactivate virus, may not protect the immunized individual from disease and in some cases may even exacerbate disease upon infection by infectious virus [9]. For example, Geeraedts et al. reported that formaldehyde (from which formalin is derived) interferes with the fusion ability of the inactivated influenza A virus particle [10]. Fusion mediated binding to cell endosomes is essential for TLR7-mediated IFN-α induction necessary for stopping further virus infection. Inhibiting membrane fusion by inactivated virus vaccine due to formalin destruction of fusion proteins would render the vaccine ineffective. If this is so, then virus inactivation procedures that compromise fusion activity of inactivated whole virus vaccines, like formaldehyde treatment, could potentially reduce or totally eliminate vaccine efficacy. Another problem is that formalin is toxic. It needs to be neutralized or removed from the vaccine preparation [9].

β-propiolactone is also commonly used as a virus inactivation agent to produce inactivated whole virus vaccines and is not as problematic as formalin for making inactivated whole virus vaccines. In contrast to formalin, β-propiolactone does not need to be removed from inactivated virus preparation, since it is rapidly hydrolyzed [9]. However, β-propiolactone, as does formalin, can readily interact with nucleophilic sites on amino acids and proteins of virus to be inactivated [11]. Thus, β-propiolactone could induce conformational changes on the viral surface resulting in alteration of epitopes necessary for induction of neutralizing antibodies against a pathogen [11]. Again, the vaccine would be rendered useless. In addition, β-propiolactone has also been recognized as a carcinogen, but apparently it is not carcinogenic at the concentrations used to inactivate viruses [9].

Thus, alternative inactivating agents that are less toxic and that do not need a process to remove the inactivating agent to create an efficacious vaccine should be evaluated as possible agents to make inactivated whole virus vaccines.

Therefore, a grape seed extract designated as Galahad™ (Patent: US8629121B2) was evaluated for inactivation of multiple viruses. The Galahad™-inactivated influenza preparation was developed as an intranasal vaccine to achieve vaccine efficacy against Influenza A H5N1 virus. This vaccine needs no preservative for storage to be efficacious.

The hypothesis to be tested was that mice would be protected from a lethal influenza virus infection when immunized with Galahad™-treated virus.

## 2 Materials and methods

### 2.1 Extraction and preparation of Galahad™

Many sources of grape seed from various growing seasons were used over time to extract pure, consistent batches of pure Galahad™. Various concentrations of Galahad™ in distilled water were prepared from 5% grape seed extract (supplementary section IV, 1.1). For cell culture experiments and in vivo vaccine experiments, Galahad™ was filtered through a micropore filter (0.2-micron, Millipore Sigma, St. Louis, MO, USA) to provide material free of bacterial contaminants. Methods for characterizing Galahad™ are found in supplementary section [IV-IX]. Final product of each batch was tested for concentration using a photospectrometer (Genesys 10S UV-Vis) at 439NM (linear equation y = 0.5900995 x -8.583618E-03).

### 2.2 Cells and viruses

#### 2.2.1 Cells

Vero 76 cells from American Type Culture Collection (ATCC, Manassas, VA) were grown in minimal essential medium (MEM) from Thermo Fisher Scientific-Gibco (thermofisher.com, Fisher Scientific, Waltham, MA, USA) supplemented with 5% fetal bovine serum (FBS) from Thermo Fisher Scientific (Logan, UT) and 0.1% NaHCO_3_. Human lung carcinoma cells (A-549) were obtained from ATCC and were grown in Dulbecco's MEM (Gibco BRL) supplemented with 0.1% NaHCO_3_ and 10% FBS. Madin-Darby canine kidney cells (MDCK) were obtained from American Type Culture Collection (ATCC, Manassas, VA, USA).

#### 2.2.2 Viruses

Adenovirus 1 (Chicago) was obtained from World Reference Center for Emerging Viruses and Arboviruses (WRCEVA). This virus was propagated in Vero 76 cells. The medium used to create virus stocks for Vero 76 cells was minimal essential medium (MEM) supplemented with 2 mM L-glutamine, 2% FBS, 0.1% NaHCO_3_, and 50 μg/ml gentamicin. USA-WA1/2020 was obtained from WRCEVA and also propagated in Vero 76 cells as above. The SARS-CoV-2 study was done at Utah State University ABSL-3+ enhanced laboratory approved for select agent usage. Influenza A H5N1 (Duck/MN/1525/81) obtained from Dr. Robert Webster of St. Jude Hospital, Memphis, was prepared in MDCK cells. Medium used to prepare influenza virus stocks was MEM without serum, 0.18% NaHCO_3_, 20 μg trypsin/ml, 2.0 μg EDTA/ml, and 50 μg gentamicin/ml.

This influenza A H5N1 virus was adapted to mice by passaging it through mice until virus induced pneumonia-associated death in mice exactly as described by Sidwell et al [12].

Viruses in samples were quantified using the method described by Reed and Muench [13]. Cell culture media used for quantifying Adenovirus 1 or SAR-CoV-2 was MEM supplemented with 2% FBS and 50 μg/ml gentamicin. For Influenza A H5H1 virus quantification, MEM without serum, 20 μg trypsin/ml, and 2.0 μg EDTA/ml was used. All virus titers are expressed as TCID_50_ units (50% tissue culture infectious dose).

### 2.3 Animals

For the seven-day toxicity study, approximately 42-day-old male and female Sprague Dawley rats were obtained from Harlan Sprague Dawley, Inc. Animals were housed one animal per cage during the study.

For vaccine studies, specific pathogen-free 18–21 g (5–6 weeks old) BALB/c mice (female) were obtained from Charles River Laboratories (Wilmington, MA).

#### 2.3.1 Ethics regulation of laboratory animals

These studies were conducted in accordance with approval of Institutional Animal Care and Use Committees at various institutions where experiments were done. All are AAALAC-accredited laboratories. Animal experiments were also done in accordance with the National Institute of Health Guide for the Care and Use of Laboratory Animals (Assurance no. A3801-01).

### 2.4 Virucidal efficacy evaluation

Influenza A H5N1 virus was exposed to an equal volume of six ½ log_10_ dilutions of Galahad™ or PSS for 10 min at room temperature. Virus was titered as described above.

Representative viruses from other genera, including SARS-CoV-1 and SARS-CoV-2 were also treated in the same manner as above with various dilutions of Galahad™ for 10 min (See Supplementary Tables S5–S7).

### 2.5 Electron microscopy

Non-infectious adenovirus (Ad5-CMV-empty) was obtained from Baylor College of Medicine Vector Development Laboratory (Houston, Texas, USA). Influenza virus-like particles (VLPs) were prepared from *T. ni* pupae as described in the supplementary section V [14]. Galahad™ (∼68 kDa at a concentration of 40 mg/ml) was diluted 100-fold or 250-fold with HEPES-NaCl-CaCl_2_ buffer (20 mM N-[2-Hydroxyethyl] piperazine-N'-[2-ethanesulfonic acid], 150 mM NaCl, 0.11 mM CaCl_2_, pH 7.6).

Adenovirus particles were mixed 1:1 with HEPES-NaCl-CaCl_2_ buffer or with 250-fold diluted Galahad™. After mixing, particles mixed with buffer were prepared as a negatively stained specimen. Particles mixed with diluted Galahad™ were incubated at room temperature for 2, 12, 47, and 240 min before being prepared as negatively stained specimens.

Influenza VLPs were mixed 1:1 with HEPES-NaCl-CaCl_2_ buffer and incubated for 6 min. Influenza VLPs were also mixed with 100-fold diluted Galahad™ and allowed to incubate for 1, 2.5, 12, and 42 min. After each incubation period, sample was prepared as a negatively stained specimen.

Specimens were imaged via negative-stain transmission electron microscopy. To prepare each negatively stained specimen, 3.5 μL of sample was withdrawn and placed on a glow-discharged Formvar/C coated grid. After incubation of 0.5–1 min, grid was blotted with filter paper and quickly placed in 20 μL of buffer and quickly removed (time in drop was about 1 second). Grid was again blotted with filter paper and placed again in buffer, withdrawn, and blotted. This last step was repeated using a 20 μL drop of 1% uranyl acetate or 1% ammonium molybdate (negative-stain solutions) instead of buffer. After blotting, grid was placed in another 20 μL drop of the same negative-stain solution for 15–20 seconds. Finally, grid was blotted with filter paper and allowed to air dry. All 20 μL drops were placed on Parafilm. Specimens were imaged in a ThermoFisher Tecnai 12 transmission electron microscope. Images were recorded on a Gatan Ultrascan digital camera.

### 2.6 Seven-day toxicity study

Sprague Dawley rats were observed and recorded each day along with the temperature and humidity of the animal room. Five males and five females were used for the toxicity study. Animals were dosed once using one of five concentrations of Galahad™. Doses used were 0.5 (undiluted Galahad™), 0.25, 0.125, 0.0625, and 0.03125 ml per animal. Lower concentrations were prepared by subsequent dilution in 0.9% NaCl for injection (USP). Galahad™ was administered intravenously at dosing volume of 0.5 ml per administration. Animals receiving undiluted Galahad™ were observed for 30 min before dosing animals receiving lesser concentrations of Galahad™. All animals were observed for seven days for clinical signs and symptoms of toxicity. On day eight after dosing with Galahad™, animals were euthanized by CO_2_ asphyxiation then the body cavity was opened and each organ was visually inspected for abnormal morphology of organs. Body weights of animals were recorded prior to dosing and on the eighth day, before gross necropsy was done.

### 2.7 Vaccine formulation

Influenza A H5N1 virus (MN/1525/81) using a tissue culture infectious dose assay (50% tissue culture infective dose = TCID_50_) was diluted to 10^5.7^ TCID _50_/ml, 10^4.7^ TCID _50_/ml, or 10^3.7^ TCID_50_/ml in MEM. These preparations were treated with equal volume of undiluted Galahad™ or PSS for 10 minutes or 24 hours at 37 °C. To stop further degradation of the virus due to temperature and likely to the exposure to Galahad™ inactivation of virions, preparations were aliquoted and stored at –80 °C until used to immunize animals.

Mice were inoculated with virus preparation in which virus had been exposed to Galahad™ for 10 minutes. Animals received one dose of this vaccine intranasally and the preparation was designated as Vaccine 1. Currently, inactivated vaccines generally require two doses at 0 and 14 days, 0 and 21 days, or 0 and 28 days (See CDC guidelines for immunization schedules.) although some polysaccharide capsular vaccines use a 14-day interval between immunizations [15]. The 14-day interval was chosen since it was not known when antibody response to first immunization would wane. Thus, the preparation was given to mice twice intranasally (immunizations 14 days apart) and was designated as Vaccine 2. Another aliquot of virus was exposed to Galahad™ for 24 hours and the preparation was designated as Vaccine 3. Mice receiving Vaccine 3 received two immunizations intranasally, given 14 days apart. For each vaccine trial, all mice were challenged 14 days after the last immunization with homologous infectious H5N1 Influenza A virus (10^5^ TCID_50_/ml). PSS acted as a control.

### 2.8 Experimental design for assessing vaccine efficacy

Twenty mice per group were immunized once or twice, intranasally, with various concentrations of Galahad™-inactivated influenza A H5N1 Duck/MN/1525/81, inactivated for 10 minutes or for 24 hours. Mice were individually weighed prior to each vaccine or PSS immunization and then on day of virus challenge and subsequently on days three, six, and on day 10 or day 14 after virus challenge to determine average weight change for all animals in each treatment group. On days three and six, and on day 10 or day 14 after virus challenge, each lung of a surviving mouse was weighed, and the lung was set aside for examination of pathology and for determining virus lung titers. Animals that lost greater than 30% of their initial body weight or were extremely moribund were humanely euthanized by CO_2_ asphyxiation, and day of euthanasia was designated as day of death due to infection.

### 2.9 Neutralizing antibody assay

An equal volume of a serum sample (diluted 1/100) was mixed with virus (Influenza A H5N1 (Duck/MN/1525/81) with a titer of 200 TCID_50_/ml and incubated at 37 °C for 1 hour. This preparation was serially diluted and the surviving virus titered by CPE (cytopathic effect) assay. Eight dilutions were plated in quadruplicate and assay was done three times on the same plate for each serum sample. For CPE assay, 0.1 ml of neutralized virus was added directly to cell culture plate containing MDCK cells plated the previous day in a 96-well plate. An additional 0.1 ml of medium, containing 20 μg trypsin/ml, 2.0 μg EDTA/ml, and 50 μg gentamicin/ml (all final concentrations) was then added to each well, gently mixed and incubated at 37 °C for 6 days, the optimal time required to achieve full cytopathic effect in the non-treated infectivity controls when using virus at 200 TCID_50_ units. Wells in the plate were then scored by visual observation for cytopathic effect or cytotoxicity using light microscopy. CPE was graded upon a scale of 0–4; 0 = no cytopathic effect and 4 = 100% cytopathic effect. Titers were then calculated using Reed-Muench method [13]. The inverse of the most dilute serum sample completely protecting cells from virus cytopathic effects was considered virus neutralization titer for the serum.

### 2.10 Lung virus titer determination

At day three, day six, or day 14, each mouse lung was homogenized in 1 ml of MEM solution and assayed in triplicate for infectious virus in Madin-Darby canine kidney cells (MDCK), as described previously [16]. Samples from each test group were pooled and titered in duplicate and titers compared to titers of samples from untreated controls.

### 2.11 Methods of lung pathology determination

#### 2.11.1 Lung scoring

At day three, day six, or day 14, each mouse lung lobe was removed, weighed, placed in a petri dish, and then assigned a score ranging from 0 (normal appearing lung) to 4 (maximal plum coloration in 100% of the lung).

#### 2.11.2 lung function

Lung function was evaluated by measuring arterial saturated oxygen levels (SaO_2_) of each animal from days four to eight after exposure to virus. For these studies, SaO_2_ measurements were made using MouseOx™ (STARR Life Sciences, Pittsburgh, PA) pulse oximeter with collar attachment designed to specifically measure SaO_2_ levels in rodents. Mean SaO_2_ levels were calculated at each time period for each treatment group.

#### 2.11.3 Gross pathology scoring

On days three, six, and then day 10 or 14 after virus challenge, mice were necropsied, and gross pathology of lungs was scored. Surviving mice from each treatment group were sacrificed and lungs were scored for consolidation and for distribution of surface lesions (focal, multifocal, diffuse) and lung discoloration (red to dark purple to almost black; i.e., focal/red = score of 1, multifocal/darker red = 2; multifocal, diffuse/intense red or purple = 3) or severe hemorrhaging of entire lung (entire lung surface appearing purple or almost black = 4).

#### 2.11.4 Histopathology

After observation of gross pathology, right lobes of lungs from surviving mice from each treatment group were harvested and fixed in 10% neutral buffered formalin. Formalin fixed tissues were embedded in paraffin, sectioned at five microns per slice, stained with hematoxylin and eosin stain, and evaluated for microscopic lesions by a board-certified veterinary pathologist. Distribution, description, and severity of lesions were recorded. See representative images of lung pathology in supplementary section, Figure S3.

### 2.12 Statistical analysis

Mean day of death was calculated and analyzed by the Kruskal-Wallis test followed by Dunn's post tests for evaluating the significance of pairwise comparisons. Significant differences in lung virus titers were analyzed by one way ANOVA. Subsequent pairwise comparisons were made using Newman-Keuls post-tests. Significant differences in lung scores were determined by the Kruskal-Wallis test followed by Dunn's pairwise comparison post-tests to determine significance of the pairwise comparisons. Analysis of significant differences in SaO_2_ levels were analyzed by the Kruskal-Wallis test followed by Dunn's post-test for evaluating significant pairwise comparisons. Survival analysis was done using Kaplan-Meier method and a Logrank test. When that analysis revealed significant differences among the treatment groups, then pairwise comparisons of survivor curves (PSS vs. any treatment) were analyzed by Gehan-Breslow-Wilcoxon test, and relative significance was adjusted to a Bonferroni-corrected significance threshold for number of treatment comparisons done. All statistical analyses were performed using GraphPad Prism 6 (GraphPad Software Inc., La Jolla, CA).

## 3 Results

### 3.1 Compound characterization

Galahad™ was found to consists of two main polymers which are both needed for full biological activity. Galahad™ is composed of 90% proanthocyanidin made of catechin monomers (See Supplementary Materials, Figure S1) and 10% polysaccharide consisting primarily of eight simple sugars (See Supplementary Materials, Table S1–S4). Two peaks were detected using dynamic light scattering test. One was of molecular weight of around 1.6 million Daltons and represented 5% of Galahad™ preparation and the other representing 95% had a molecular weight of around 68,000 Da.

### 3.2 Virucidal efficacy evaluation

To determine biological effects of Galahad™ on viruses, before formulating a vaccine, studies were done to determine if Galahad™ could inactivate a wide range of RNA and DNA viruses including SARS-CoV-2 when used undiluted (standardized to 4 mg/ml) or diluted (See Supplementary Material, Tables S5–S7). It was of particular interest to determine virus inactivating effects on Influenza A H5N1 virus from which a vaccine was to be created.

#### 3.2.1 Influenza A H5N1 virus

Galahad™ treatment of Influenza A H5N1 virus led to significant reduction of virus titers at all dilutions tested (P < 0.01-P<0.001, Table 1). Treatment with PSS without Galahad™ did not reduce virus titer.

**Table 1:** Virucidal effect of Galahad™ on Influenza A H5N1 virus.

Treatment	Galahad^TM^ Concentration (%)	Virus Titer No Treatment (Log_10_ TCID_50_/0.1ml)	Virus Titer with Treatment (Log_10_ TCID_50_/0.1ml)	Log_10_ Reduction of Virus Titer
Galahad^TM^	Undiluted	3.5	0	3.5∗∗∗
Galahad^TM^	10	3.5	0	3.5∗∗∗
Galahad^TM^	0.1	3.5	1.75	1.75∗∗
PSS	-	3.5	3.5	0

∗∗P < 0.01, ∗∗∗P < 0.001.

^a^Virus titer was below detectable limits.

Representative viruses from other genera were also tested, including a strain of SARS-CoV-2. Most viruses tested were inactivated in the presence of undiluted Galahad™ or more dilute concentrations (See Supplementary Tables S5–S7). Interestingly, only undiluted Galahad™ significantly reduced virus titer.

### 3.3 Effects of Galahad™ on virus integrity evaluated by electron microscopy

**Figure 1:**
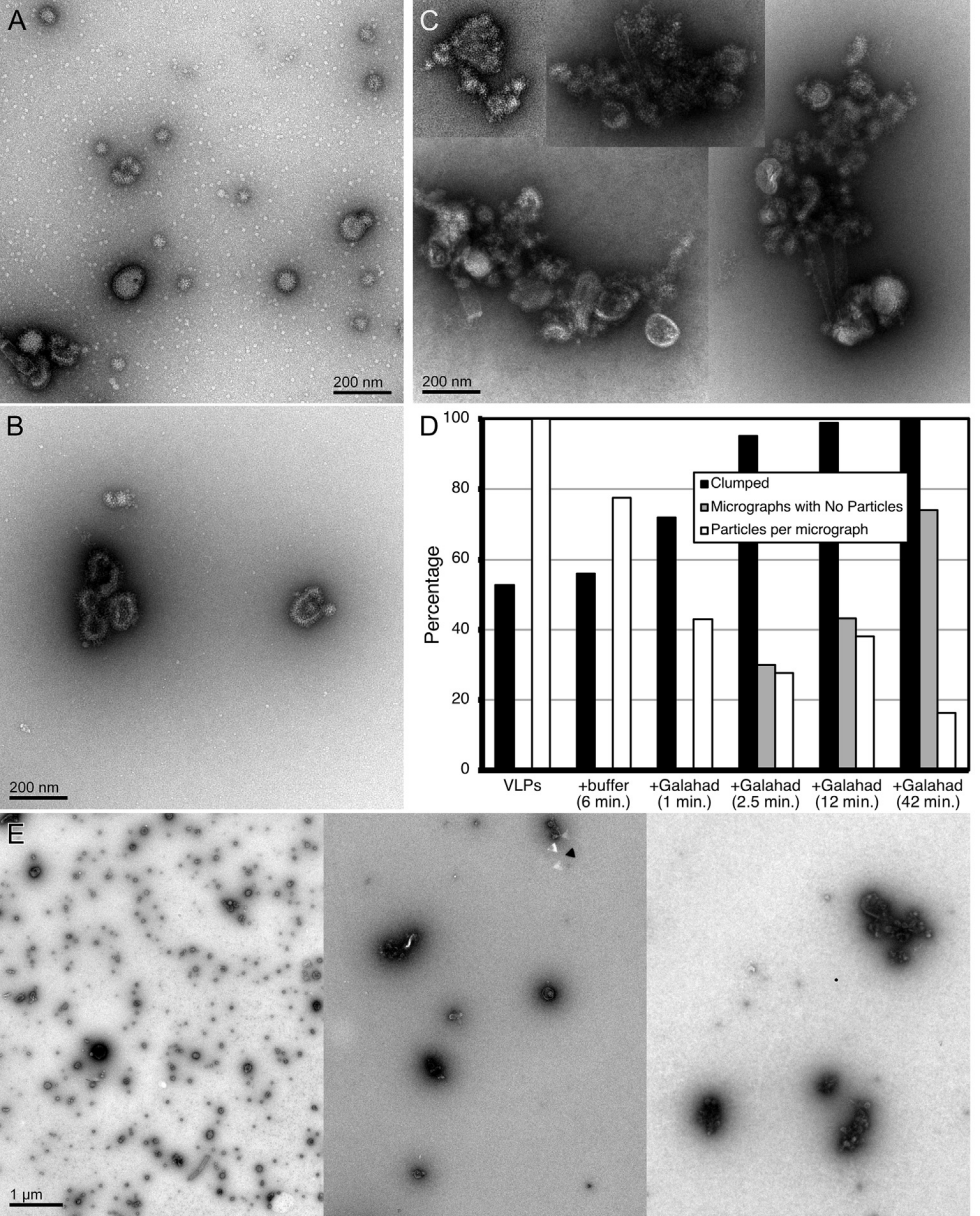
Electron microscopic examination of the effects of Galahad™ (200 μg/mL) treatment on the structure of influenza virus-like particles. A. Influenza VLPs without treatment, shown at a high magnification. B. VLPs at 2.5 minutes after exposure to Galahad™ shown at high magnification. C. VLPs at 12 minutes after exposure to Galahad™ shown at high magnification. D. Percentage of clumped particles observed (black bars), micrographs without particles (gray), and particles per micrograph compared to those in the untreated experiment (white bars). Views of particles are similar to those seen in micrographs shown in panels A–C and were used to count the particles to calculate the percentages shown in panel D. E. VLPs untreated (left), Galahad™-treated for 2.5 minutes (middle), and Galahad™-treated for 12 minutes (right), shown at a medium magnification.

Given virucidal activity discussed above, preparations of concentrated influenza VLPs and adenovirus virions were examined by electron microscopy to see if Galahad™ treatment had an observable effect on their morphology. Both influenza and adenovirus particles not treated with Galahad™ were distributed in a dispersed fashion (Figure 1A; Supplemental Figure S2.B panels A, C). A few virus clumps were present, but were typically small. After brief exposure to Galahad™ (1–2.5 minutes), more clumping was observed for both influenza and adenovirus VLPs (Figure 1B, D; Figure S2.B panels B, D). After longer exposure (12–240 minutes), few, if any, influenza and adenovirus VLPs remained unclumped (Figure 1C, D; Figure S2.B panels C, D). As exposure time increased, more clumps of viruses were observed. At the longest periods of time, most of the clumped particles were no longer detectable. After 240 minutes, very few clumped or unclumped adenovirus were detectable compared to untreated adenovirus (Figure S2A; B) and similarly after 42 minutes for influenza VLPs (Figure 1D; Figure S2C). In both cases, as seen in medium or low magnification views, untreated particles had a punctate appearance (Figure 1E; Figure S2A; B; C). Galahad™ induced coalescing of virions into large spots or clumps at initial and medium time points for adenovirus (Figure S2. A; 2, 12, and 47 minutes) and for influenza VLPs (Figure 1 B, C, E; Figure S2C). At the end points of exposure for both, debris was primarily observed (Figure S2A, 240 minutes and Figure S2C, 42 minutes) indicating that virion integrity was destroyed.

### 3.4 Seven-day toxicity study in rats

It was critical to determine the toxicity of Galahad™ for formulating a vaccine. Thus, a seven-day study in rats was undertaken. Mean body weights of animals treated with various dilutions of Galahad™ is shown in Table 2. Mean body weight for male and female rats increased throughout the experiment. Other clinical signs or symptoms of toxicity were also evaluated including unusual appearance of the animals, unusual body secretions, and abnormal behavior. None of these clinical signs or symptoms of toxicity were observed.

**Table 2:** Seven-day toxicity study of Galahad™ treatment of rats.

Sex of Animal	Dose Levels (mL/Mouse)	Body weights on Day 1^a^	Body weights on Day 8	% Change in Body Weight
Male	0.50	246	295	19.9
	0.25	242	295	21.9
	0.125	244	300	23.0
	0.0625	246	296	20.3
	0.03125	239	283	18.4
Female	0.50	155	169	9
	0.25	157	173	10.2
	0.125	162	182	12.3
	0.0625	164	192	17.1
	0.03125	160	180	12.5

a Body weights were taken prior to dosing with Galahad™.

### 3.5 Vaccine evaluation

Since the seven-day toxicity study data indicated that Galahad™ is very likely not toxic, vaccines were formulated to test the hypothesis that Galahad™-inactivated influenza A virus could be an effective vaccine to protect mice against influenza A H5N1 virus infection.

#### 3.5.1 Efficacy of vaccine 1

**Figure 2:**
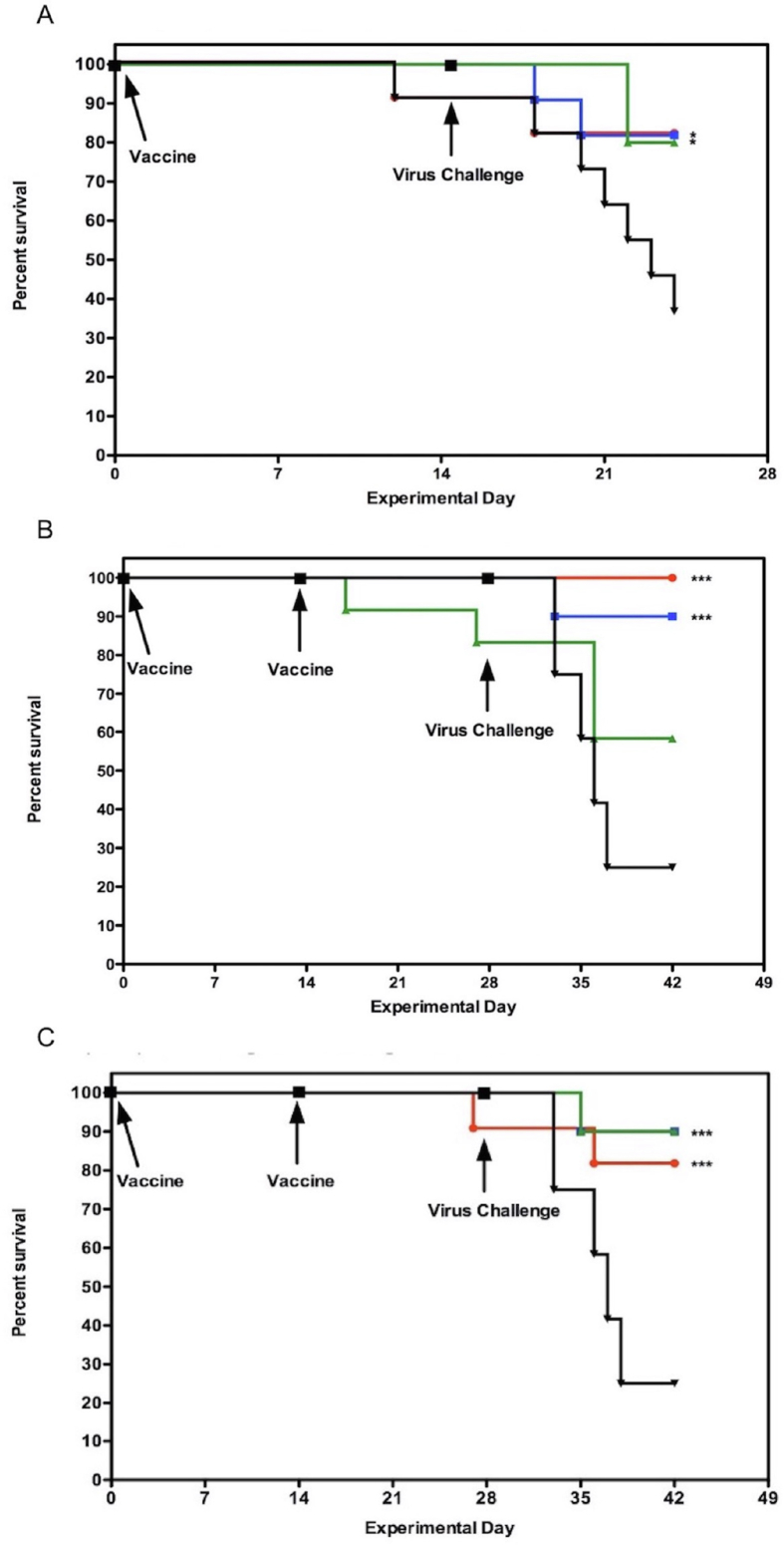
Survival of BALB/c Mice Immunized with Galahad™ Inactivated Vaccines. A. Vaccine 1: 10-minute exposure to Galahad™ with vaccine given once intranasally to BALB/c mice and subsequently challenged with 10^3.5^ TCID50 influenza A H5N1 (Duck/MN/1525/81). 10^5.7^ TCID_50_∗P < 0.05. 10^4.7^ TCID_50_ ∗P < 0.05. 10^3.7^ TCID_50_ ∗P < 0.05. No Vaccine (PSS). B. Vaccine 2: 10-minute exposure to Galahad™ with vaccine given twice intranasally to BALB/c mice and subsequently challenged with 10^3.5^ TCID50 influenza A H5N1 (Duck/MN/1525/81). 10^5.7^ TCID_50_ ∗∗∗P < 0.001. 10^4.7^ TCID_50_ ∗∗∗P < 0.001. 10^3.7^ TCID_50._. No Vaccine (PSS). C. Vaccine 3 a 24-hour exposure to Galahad™ with vaccine given twice intranasally to BALB/c mice and subsequently challenged with 10^3.5^ TCID50 influenza A H5N1 (Duck/MN/1525/81). Mice received vaccine 2 and 3 at 28 and 14 days before virus challenge; vaccine 1 14 days before virus challenge. 10^5.7^ TCID_50_ ∗∗∗P < 0.001. 10^4.7^ TCID_50_ ∗∗∗P < 0.001. 10^3.7^ TCID_50_. No Vaccine (PSS).

When Vaccine 1 was delivered once intranasally, all virus dilutions of inactivated virus tested afforded significant protection against death; up to 80% of immunized mice in each vaccine group survived (Figure 2, Panel A) (p < 0.05). Two courses of Vaccine 1 at 10^5.7^ TCID_50_/ml and 10^4.7^ TCID_50_/ml (Galahad™-inactivated for 10 minutes) delivered intranasally significantly protected mice from death, although Vaccine 1 at 10^5.7^ TCID_50_/ml prevented death in all immunized mice that survived to the time of challenge and Vaccine 1 at 10^4.7^ TCID_50_/ml protected 80% of the animals from death (Figure 2, Panel A) (P < 0.05). One animal receiving Vaccine 1 at 10^5.7^ TCID_50_/ml and a placebo mouse were found dead 3 days before virus challenge of unknown causes. Rigor mortis and autolysis made examination impossible. One mouse receiving Vaccine 1 at 10^5.7^ TCID_50_/ml and placebo-treated mouse were found dead 3 days post virus challenge. Again rigor mortis and autolysis made examination impossible. However, animals receiving Vaccine 1 at 10^3.7^ TCID_50_/ml delivered once intranasally did not effectively protect mice against virus challenge, with only 60% of those animals surviving. In this vaccine trial arm, 60% of the mice receiving no vaccine died. The mean day of death was similar for animals that died in each group, although considerably more mice died in the unimmunized group of mice (Table 3).

**Table 3:** Mean day of death.

Vaccine Dose^a^	Vaccine 1	Vaccine 2	Vaccine 3
10^5.7^	3.0 ± 0.0^b^	>14∗	8.0 ± 0.0^d^
10^4.7^	6.0 ± 0.0	5.0 ± 0.0	7.0 ± 0.0
10^3.7^	8.0 ± 0.0	6.8 ± 1.8^c^	7.0 ± 0.0
PSS	6.3 ± 2.3^b^	6.4 ± 1.4^c^	6.4 ± 1.4

∗P < 0.05.

a Titers are expressed as TCID_50._ units for infectious virus titers before inactivation with Galahad™.

b One animal receiving Vaccine 1 at 10^5.7^ TCID_50_ was found dead 3 days before virus challenge of unknown causes and another mouse in this group died at day 3 post virus challenge. Rigor mortis and autolysis made examination of the second mouse impossible. One animal receiving the placebo in the Vaccine 1 group was found dead 3 days before virus challenge of unknown causes.

c One animal receiving Vaccine 2 at^3.7^ TCID_50_ was found dead at day 7 before virus challenge of unknown causes. One mouse died at day 0 after virus challenge. Rigor mortis and autolysis made examination of this mouse impossible. It is presumed that it died due to trauma of the injection of challenge virus.

d One animal receiving Vaccine 3 at^5.7^ TCID_50_ was found dead after virus challenge. Rigor mortis and autolysis made examination of this mouse impossible. It is presumed that it died due to trauma of the injection of challenge virus.

**Figure 3:**
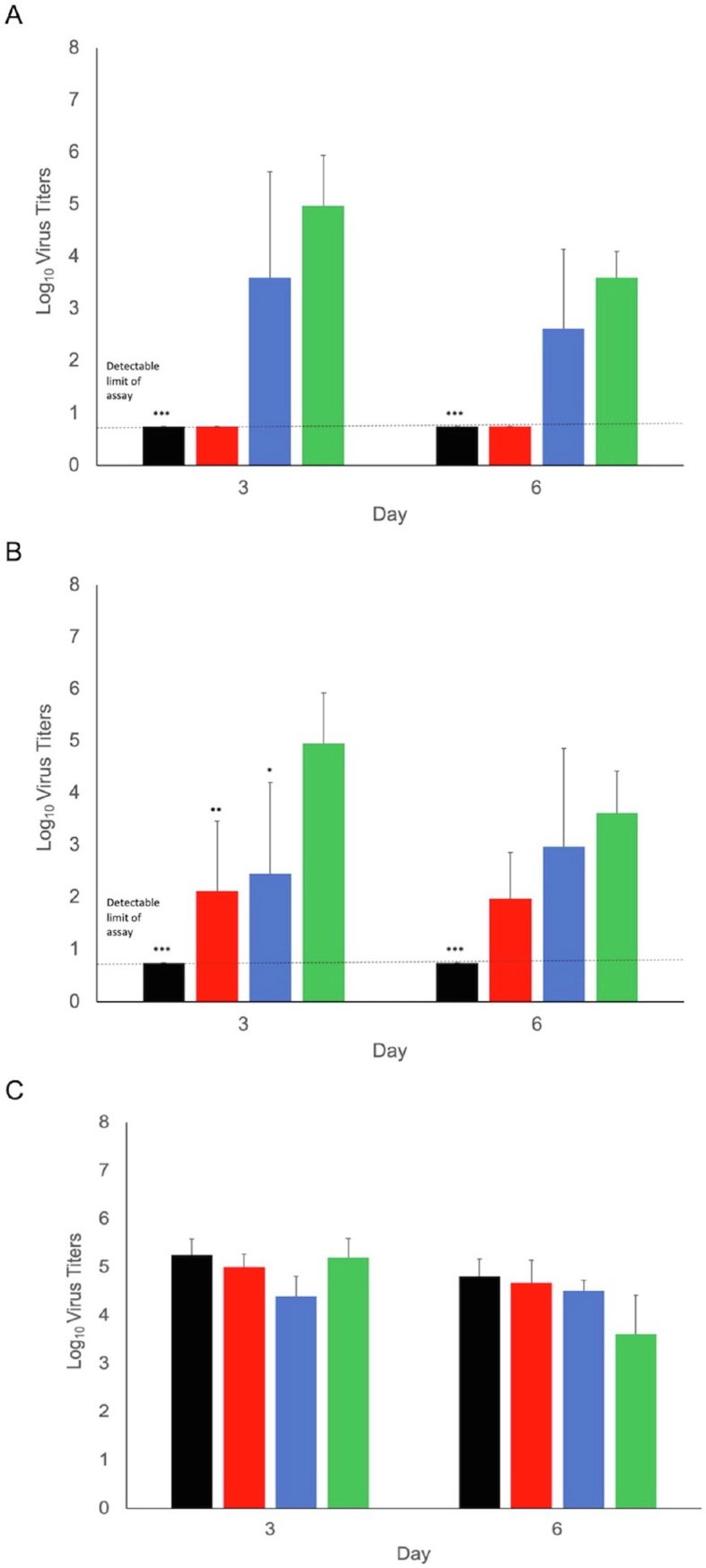
The Effect of Galahad™-Inactivated Vaccines on Lung Virus Titers after Infectious Virus Challenge. A. Vaccine 1: 10-minute exposure to Galahad™ with vaccine given once intranasally to BALB/c mice and subsequently challenged with 10^3.5^ TCID50 influenza A H5N1 (Duck/MN/1525/81). B. Vaccine 2: 10-minute exposure to Galahad™ with vaccine given twice intranasally to BALB/c mice and subsequently challenged with 10^3.5^ TCID50 influenza A H5N1 (Duck/MN/1525/81). C. Vaccine 3: 24-hour exposure to Galahad™ with vaccine given twice intranasally to BALB/c mice and subsequently challenged with 10^3.5^ TCID50 influenza A H5N1 (Duck/MN/1525/81). Mice received vaccine 2 and 3 at 28 and 14 days before virus challenge; vaccine 1 14 days before virus challenge. ^5.7^ TCID_50_ ∗∗∗P < 0.001. ^4.7^ TCID_50._^3.7^ TCID_50._No Vaccine (PSS).

Another marker of vaccine efficacy is reduction of virus lung titer. No virus was detected in mice receiving Vaccine 1 delivered intranasally at 10^5.7^ TCID_50_/ml and 10^4.7^ TCID_50_/ml (Figure 3, Panel A; day three and day 6, P < 0.001). Mice receiving Vaccine 1 at 10^3.7^ TCID_50_/ml or placebo had similar amounts of virus in lungs at both days three and six.

**Figure 4:**
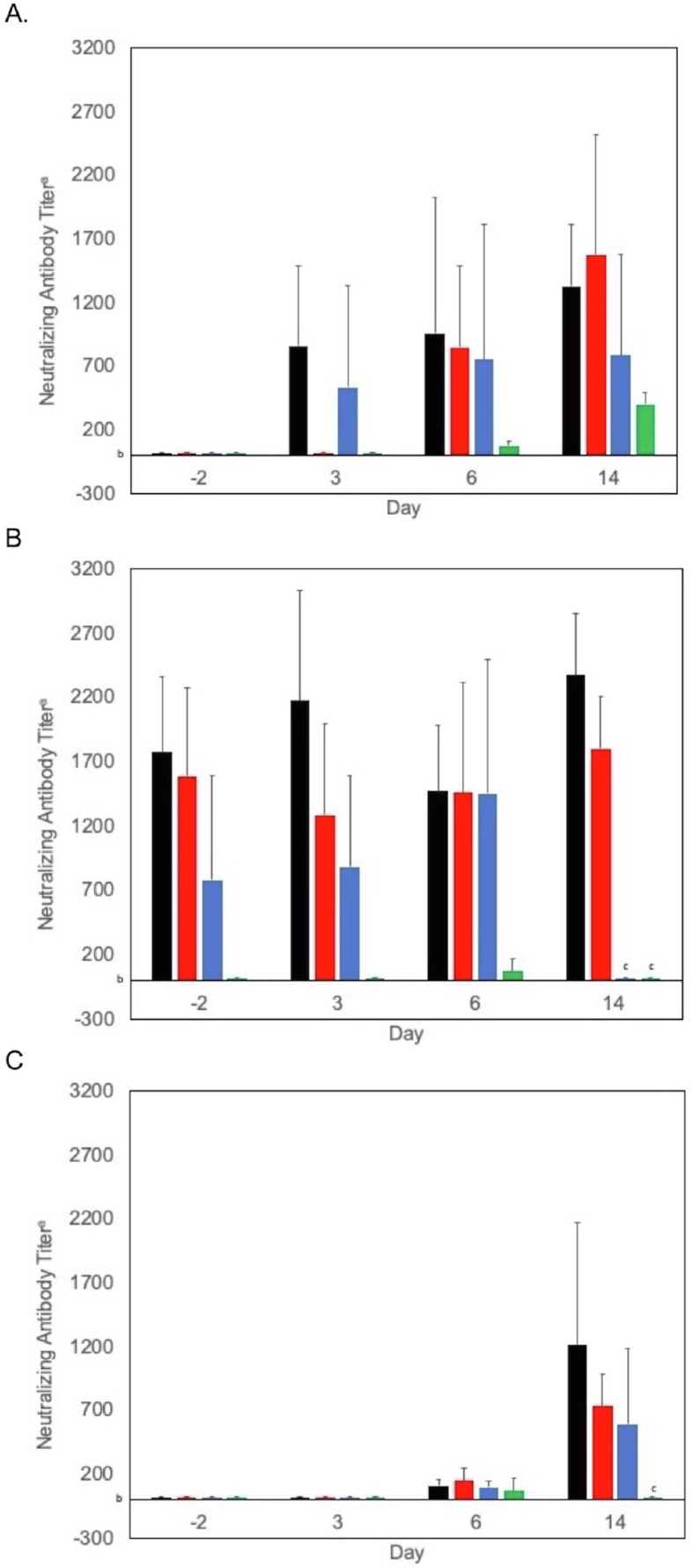
Virus Neutralizing Antibody Titers Measured Relative to Time of Infectious Virus Challenge (10^3.5^ TCID50). A. Vaccine 1: 10-minute exposure to Galahad™ with vaccine given once intranasally to BALB/c mice and subsequently challenged with 10^3.5^ TCID50 influenza A H5N1 (Duck/MN/1525/81). B. Vaccine 2: 10-minute exposure to Galahad™ with vaccine given twice intranasally to BALB/c mice and subsequently challenged with 10^3.5^ TCID50 influenza A H5N1 (Duck/MN/1525/81). C. Vaccine 3: 24-hour exposure to Galahad™ with vaccine given twice intranasally to BALB/c mice and subsequently challenged with 10^3.5^ TCID50 influenza A H5N1 (Duck/MN/1525/81). ^a^The inverse of the most dilute serum sample completely protecting cells from virus cytopathic effects was considered the virus neutralization titer for the serum. ^b^Detectable limit of the assay refers to lowest dilution used. Serum was diluted by a factor of 1/100 for use in the neutralizing antibody assay. ^c^Mice were so dehydrated, that it was not possible to obtain virus from samples. Mice received vaccine 2 and 3 at 28 and 14 days before virus challenge; vaccine 1 14 days before virus challenge. ^5.7^ TCID_50_. ^4.7^ TCID_50._^3.7^ TCID_50._No Vaccine (PSS).

Among several parameters that can be used to assess vaccine efficacy, one of them is the amount of neutralizing antibodies that each vaccine generates. We assayed at day three post virus challenge for neutralizing antibody and found that Vaccine 1 at 10^5.7^ TCID_50_/ml and 10^3.7^ TCID_50_/ml did elicit neutralizing antibodies at detectable levels of the assay used (Figure 4, Panel A). In contrast, placebo-treated mice and mice receiving 10^4.7^ TCID_50_/ml inactivated virus had no detectable levels of neutralizing antibody at day three post virus challenge. At day six, neutralizing antibody titers for Vaccine 1 at all dilutions tested were 10-fold greater than those detected in the lungs of placebo mice. At day 14, neutralizing antibody titers were less variable and Vaccine 1 at 10^5.7^ TCID_50_/ml and 10^4.7^ TCID_50_/ml elicited much higher neutralizing antibody titers than those detected from lungs of mice at days three and day six after virus challenge. At day 14, neutralizing antibody titers for Vaccine 1 at the two highest concentrations of inactivated virus were 3–4 times greater than those detected in lungs of placebo mice.

**Figure 5:**
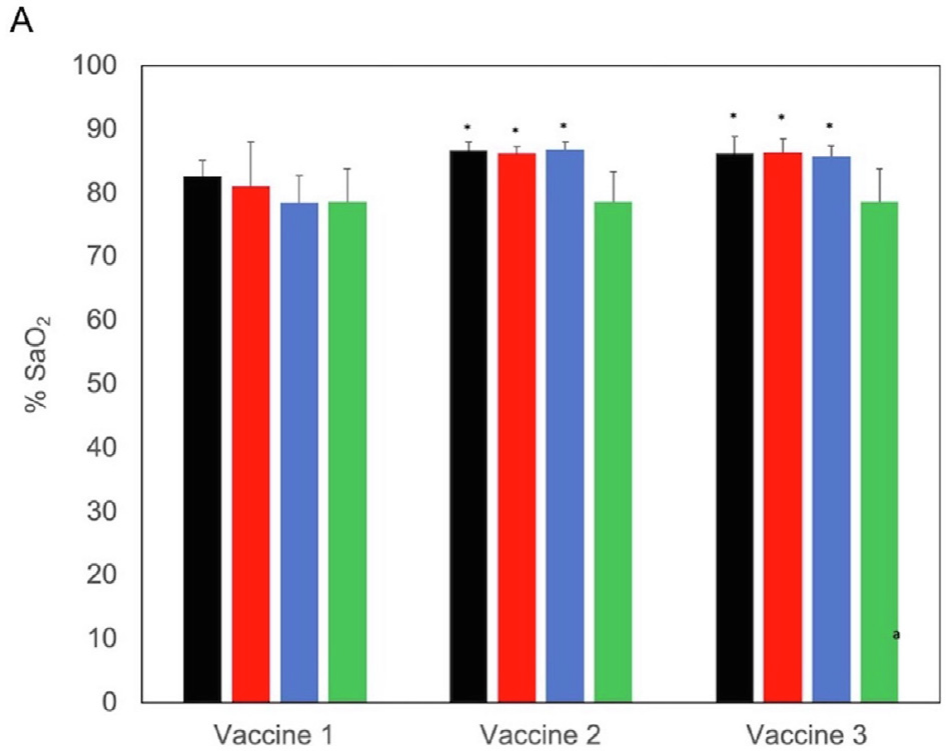
Lung Function as Measured by Saturated Oxygen (SaO_2_) Levels at Day 7 after Virus Challenge. A. Vaccine 1: 10-minute exposure to Galahad™ with vaccine given once intranasally to BALB/c mice and subsequently challenged with 10^3.5^ TCID50 influenza A H5N1 (Duck/MN/1525/81). B. Vaccine 2: 10-minute exposure to Galahad™ with vaccine given twice intranasally to BALB/c mice and subsequently challenged with 10^3.5^ TCID50 influenza A H5N1 (Duck/MN/1525/81). C. Vaccine 3: 24-hour exposure to Galahad™ with vaccine given twice intranasally to BALB/c mice and subsequently challenged with 10^3.5^ TCID50 influenza A H5N1 (Duck/MN/1525/81). Mice received vaccine 2 and 3 at 28 and 14 days before virus challenge; vaccine 1 14 days before virus challenge. ∗P < 0.05. ^5.7^ TCID_50_. ^4.7^ TCID_50._^3.7^ TCID_50._No Vaccine (PSS).

In addition to monitoring efficacy by virus yield reduction and neutralization of infectious virus, effects of virus infection were measured on lung function by monitoring lung saturated oxygen levels (SaO_2_). For current experiments, average SaO_2_ levels at day seven were slightly higher in mice receiving Vaccine at 10^5.7^ TCID_50_/ml and 10^4.7^ TCID_50_/ml compared to placebo mice (Figure 5).

**Figure 6:**
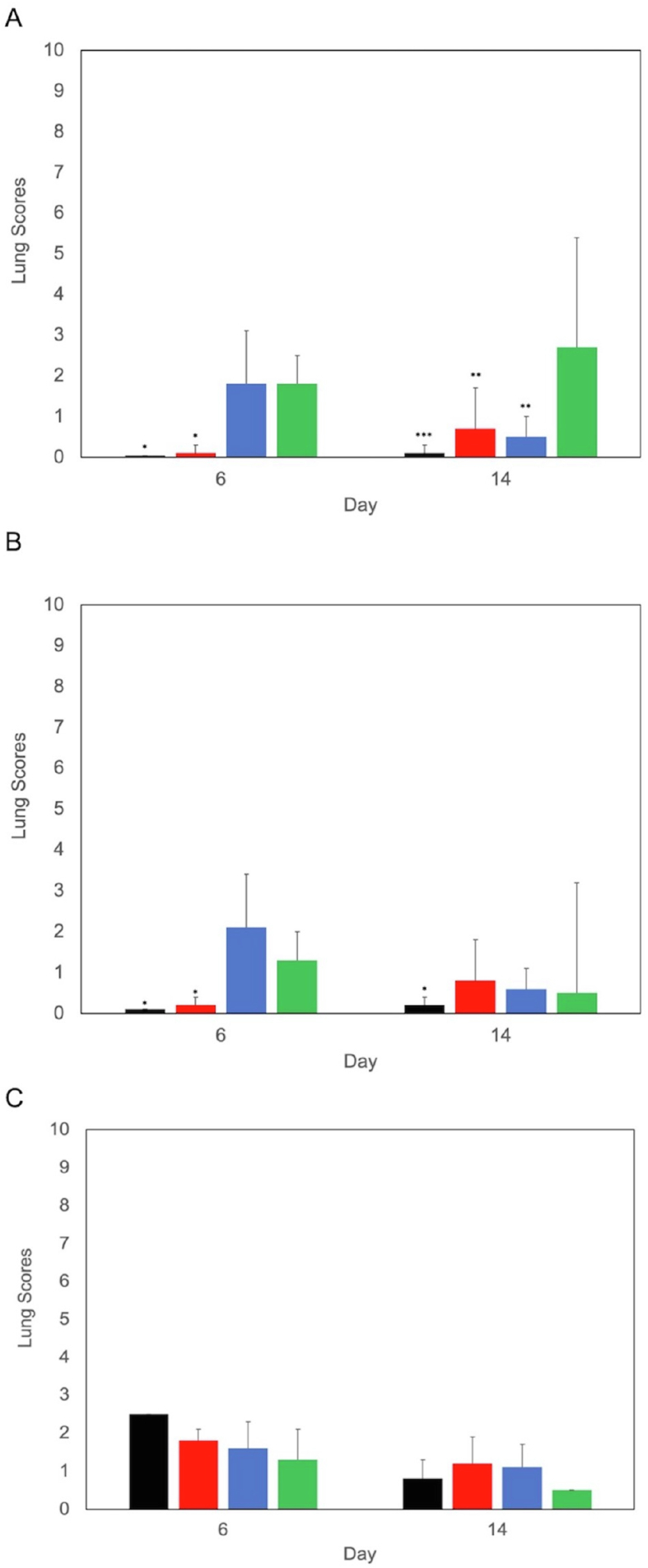
Lung Pathology Scores in BALB/c Mice Immunized with Galahad™ Vaccines A. Vaccine 1: 10-minute exposure to Galahad™ with vaccine given once intranasally to BALB/c mice and subsequently challenged with 10^3.5^ TCID50 influenza A H5N1 (Duck/MN/1525/81). B. Vaccine 2: 10-minute exposure to Galahad™ with vaccine given twice intranasally to BALB/c mice and subsequently challenged with 10^3.5^ TCID50 influenza A H5N1 (Duck/MN/1525/81). C. Vaccine 3: 24-hour exposure to Galahad™ with vaccine given twice intranasally to BALB/c mice and subsequently challenged with 10^3.5^ TCID50 influenza A H5N1 (Duck/MN/1525/81). Mice received vaccine 2 and 3 at 28 and 14 days before virus challenge; vaccine 1 14 days before virus challenge. ∗P < 0.05, ∗∗P < 0.01,∗∗∗P < 0.001. ^5.7^ TCID_50_. ^4.7^ TCID_50._^3.7^ TCID_50._No Vaccine (PSS).

Another way of detecting damage to lungs due to a virus respiratory infection is examination of lung pathology (lung scores). Lung pathology of immunized mice on day six significantly differed from the lung scores for placebo mice (P < 0.05) (Figure 6, Panel A). At day six, pathology of lungs was described as mild for all mice (Table 4). On day 14, lung scores of immunized mice were significantly different than surviving placebo-treated mice (Figure 6, P < 0.01-<0.001). Extent of pathology was described as mild for immunized mice (Table 4, see Supplementary Materials, Figure S3 for representative images of mild, moderate, severe pathology). At day 14, pathology of lungs of four surviving mice receiving placebo was described as moderate to severe.

**Table 4:** Severity of lung pathology.

Vaccine 1		Vaccine 2		Vaccine 3	
Day 6	Day 14	Day 6	Day 14	Day 6	Day 14
Mild	Very Mild	Mild	Mild	Mild-Mod	Mild-Mod
Very Mild	Very Mild	Mild	Mild	Mild-Mod	Mild-Mod
Mild	Mild	Mod	Mod	Mild-Mod	Mild-Mod
Mild	Mod-Severe	Mod	Mod	Mild-Mod	Mild-Mod

See Figure S7 for representative images of lungs demonstrating mild, moderate, or severe pathology or no pathology.

#### 3.5.2 Efficacy of vaccine 2

Eighty percent of the unimmunized mice forming the control group associated with Vaccine 2 died by day 14 after virus exposure (Figure 2, Panel B). All mice receiving Vaccine 2 (10 minute exposure to Galahad™, twice intranasally) at 10^5.7^ TCID_50_/ml survived virus challenge. Mean day of death for this group was significantly different compared to placebo treated group of mice (P < 0.05) (Table 3). No infectious virus was detected at days three and six in mice receiving Vaccine 2 at 10^5.7^ TCID_50_/ml (P < 0.001) (Figure 3, Panel B). Ninety percent of mice immunized with Vaccine 2 at 10^4.7^ TCID_50_/ml survived and only 50% of mice receiving Vaccine 2 at 10^3.7^ TCID_50_/ml survived. One mouse in this group receiving Vaccine 2 at 10^3.7^ TCID_50_/ml died 7 days prior to virus challenge of unknown causes. Another mouse in this group died on the day 0 after virus challenge, presumably from the trauma of the injection process. Virus lung titers recorded for mice receiving Vaccine 2 at 10^4.7^ TCID_50_/ml were lower at day three than those detected in placebo group of mice (Table 3, P < 0.05) as were virus lung titers for mice receiving Vaccine 2 at 10^3.7^ TCID_50_/ml (P < 0.05) (Figure 3, Panel B) but not significantly so at day six.

For mice receiving Vaccine 2, at all concentrations, there were substantial amounts of neutralizing antibodies two days prior to virus challenge and none in mice receiving no vaccine as had been expected (Figure 4). Three days after challenge with virus, neutralizing antibody was still high in mice receiving Vaccines 2 at 10^5.7^ TCID_50_/ml and 10^4.7^ TCID_50_/ml, and, in general, these titers were substantially greater at end of trial. Placebo mice were relatively unresponsive in producing neutralizing antibodies and most of these mice subsequently died.

SaO_2_ levels measured at day seven for mice immunized with Vaccine 2 had normal SaO_2_ levels and significantly so for mice receiving Vaccine 2 at 10^3.7^ TCID_50_/ml (P < 0.05) (Figure 5, Panel B).

Average gross pathology scored for lungs at day six for mice receiving Vaccine 2 at 10^5.7^ TCID_50_/ml and 10^4.7^ TCID_50_/ml differed significantly from lungs of placebo-treated mice (P < 0.05) (Figure 6, Panel B). They differed significantly at day 14 for all mice receiving Vaccine 2 at 10^5.7^ TCID_50_/ml (P < 0.01-P<0.001) (Figure 6, Panel B), Very little discoloration on the surface of lungs (data not shown) was seen for any of mice receiving Vaccine 2 at 10^5.7^ TCID_50_/ml and 10^4.7^ TCID_50_/ml (mild) (Table 4) and there was no evidence of hemorrhaging (data not shown). In contrast, mice receiving Vaccine 2 at 10^3.7^ TCID_50_/ml or placebo, extensive discoloration was seen (data not shown) and the pathology was described as moderate (Table 4, See Figure S3 for representative images of mild and moderate pathology.)

#### 3.5.3 Efficacy of vaccine 3

Although Galahad™ had been shown to inhibit all of tested viruses within 10 minutes after exposure to Galahad™ at 37 °C (See Supplemental Material, Tables S5–S7), it was thought that longer exposures might be required for regulatory approval should this technology be approved for use. Therefore, we evaluated the efficacy of Vaccine 3 in which virus was exposed to Galahad™ for 24 hours. Mice receiving Vaccine 3 were immunized intranasally twice. Doses were given 14 days apart. Over eighty percent of mice receiving Vaccine 3 survived, which differed significantly from survival numbers recorded for placebo group of mice (P < 0.05) (Figure 2C, Panel C). When deaths did occur, they occurred on average at days seven to eight for immunized mice groups (Table 3). Virus lung titers were very similar for all groups of mice regardless of treatment group and day that samples were taken for analysis (Figure 3, Panel C).

Mice immunized with Vaccine 3 produced no detectable neutralizing antibody at day two before virus challenge nor at three days after infectious virus exposure (Figure 4, Panel C). At day six post virus challenge, the neutralizing antibody titer that was detected in mice in any vaccine group was indistinguishable from levels detected in unimmunized animals. At day 14, all doses of Vaccine 3 elicited a 4.8 to 11-fold greater neutralizing antibody response compared to neutralizing antibody response at day six.

When quantitating lung function by measuring SaO_2_ levels at day seven post virus exposure, levels of SaO_2_ of mice receiving any version of Vaccine 3 significantly differed from SaO_2_ levels measured for unimmunized group of mice (P < 0.05) (Figure 5, Panel C). Lung scores for mice receiving any Vaccine 3 dose did not differ significantly from placebo group (Figure 6, Panel C) and description of pathology for Vaccine 3 groups of mice ranged from mild to moderate at day six and day 14 (Table 4 and see Figure S3 (Supplementary Materials) for representative images of mild and moderate pathology). This suggests that maintaining appropriate lung function may not be necessarily dependent on reduction of virus titers but may be dependent on eliciting little or no lung pathology in response to virus infection.

However, Vaccine 3 did not appear to ameliorate pathology induced by virus infection when compared to unimmunized control animals when lungs were scored for pathology (Table 4).

Of the three vaccines tested, it appears vaccine 2 in which infectious virus was inactivated for 10 minutes and given to mice twice intranasally (immunizations 14 days apart) was more protective than Vaccine 1 and Vaccine 3. Lung pathology scores, lung virus titers, and production of neutralizing antibody were all much better when mice were immunized with Vaccine 2. When evaluating Vaccine 1, which was administered once intranasally and Vaccine 2 which was administered twice, both vaccines protected mice from death at equal rates. However, there were no deaths in mice receiving the highest concentration of Vaccine 2 as opposed to mice receiving Vaccine 1 in which two mice died at the highest concentration administered. More importantly, Vaccine 2 elicited a greater neutralizing antibody response, even before virus challenge. Titers were consistently high at all times that antibody responses were monitored.

## 4 Discussion

The most effective vaccine and immunization regimen evaluated for H5N1 was Vaccine 2 at 10^5.7^ TCID_50_/ml inactivated virus (Galahad™ for 10 minutes), delivered twice intranasally, with second dose coming 14 days after the primary vaccine dose. Vaccine 3 in which virus was inactivated for 24-hour, was also very effective and suggests that even when the virions were destroyed there were still highly immunogenic components remaining. A 24-hour inactivation time would likely lead to increased inactivation compared to a 10-minute exposure and perhaps lead to a safer product.

That the intranasal route of administration was an effective route of administering vaccine is in harmony of the findings of Takeda et al. [17]. They found that intranasal vaccination induced systemic antibody responses which protected mice from lethal H5N1 virus challenge.

Vaccine 3, in which virus was exposed for 24-hours, was the least effective vaccine in eliciting neutralizing antibodies before or early after virus challenge. Electron microscopy indicated that intact virions were no longer detectable after exposure to Galahad™ for even several minutes (Figure 2). Infectivity of each virus was not detectable at 5 minutes exposure to Galahad™ [data not shown]. We postulate that a 24-hour exposure time altered or destroyed the structure of three-dimensional shape of epitope(s) that would be necessary for eliciting a strong antibody response (even aggregated virions were detected at 10 minutes after exposure).

Conceivably, clumping of virions after exposure to Galahad™ may disrupt virus attachment by Galahad™ interacting directly with viral components that allow viruses to bind to a target cell. Perhaps Galahad™ “glues” virions together making clumps of virus too large to attach or prevents conformational changes necessary for attachment. Yet the vaccine still elicited an immune response, even though virions were clumped (See Fig.1 B and C). This hypothesis is supported by the finding that treating infectious influenza virus with poly-galloyl glucose (PGG) clumped virions by binding two HA trimers together via conserved receptor binding domains of HA, and this prevented virus entry [18].

Thus, Galahad™-inactivated vaccines may be efficient in producing a protective response. They are especially effective when delivered twice intranasally, a route of administration that has been shown to provide protective immunity against H5N1 influenza virus [19]; and Galahad™ has a component that inactivates virus.

Finally, there are some potential comparative advantages of a Galahad™-inactivated viral vaccine. 1) Vaccine production can be rapid (days) and simple to formulate (just add enough Galahad™ to inactivate for a given period of time, as little as 10 minutes). 2) Galahad™ is also inexpensive, likely costing pennies per vaccine. 3) Galahad™ is stable in liquid for years without special storage or longer in dried form. 4) There is a very large source of raw material available and batches can be reliably standardized to one concentration.

## 5 Summary

After only a 10-minute exposure to Galahad™, intranasal delivery of inactivated vaccine twice, 14 days apart, provided the most protective response against death, pathology, and virus infection by challenge of the H5N1 virus. An adjuvant was not used. The Vaccine 2 formulation also elicited relatively higher amounts of neutralizing antibodies than Vaccines 1 and Vaccines 3. Efficacy seen with this Galahad™-inactivated vaccine was achieved with two administrations of vaccine. Galahad™ not only inactivates viruses, but Galahad™ inactivated viruses could be used to formulate vaccines.

## 6 Conclusions

Intranasal administration of a Galahad™-inactivated influenza vaccine was an effective mode of delivery of this vaccine. A 10-minute exposure of a strain of H5N1 influenza virus A to Galahad™ is enough to inactivate infectious influenza virus to undetectable levels in the preparation to be used as the vaccine, yet it still elicited a protective immune response. Thus, Galahad™ represents an innovative way of using a catechin based molecule to derive a vaccine. This technology should be investigated further for potential clinical use in humans.

## Declarations

### Author contribution statement

Dale L Barnard, Ph.D.; David M. Belnap, Ph. D; Parastoo Azadi, Ph. D: Conceived and designed the experiments; Performed the experiments; Analyzed and interpreted the data; Wrote the paper.

Christian Heiss, Ph. D: Performed the experiments; Analyzed and interpreted the data; Wrote the paper.

D. Scott Snyder, Ph. D: Performed the experiments.

Susan C. Bock, Ph. D: Contributed reagents, materials, analysis tools or data; Wrote the paper.

Thomas W. Konowalchuk, MD: Conceived and designed the experiments; Contributed reagents, materials, analysis tools or data; Wrote the paper.

### Funding statement

Dr. Parastoo Azadi was supported by U.S. Department of Energy, Office of Science, Basic Energy Sciences [DE-SC0015662].

### Data availability statement

Data will be made available on request.

### Declaration of interests statement

The authors declare the following conflict of interests: Thomas Konowalchuk owns shares of Galaxy Force Technologies, LLC that has patent rights to produce vaccines using Galahad^TM^. The other authors declare that they have no known competing financial interests or personal relationships that could have appeared to influence the work reported in this paper. Research on Galahad^TM^ did not receive any specific grant from funding agencies in the public, commercial, or not-for-profit sectors, but was financed by companies with ownership interest in Galahad^TM^ without role in experimental design, data analysis, collection and interpretation.

### Additional information

No additional information is available for this paper.

## References

[1] Asbaghi O., Nazarian B., Reiner Ž. (2020). The effects of grape seed extract on glycemic control, serum lipoproteins, inflammation, and body weight: a systematic review and meta-analysis of randomized controlled trials. Phytother Res..

[2] Fine A.M. (2000). Oligomeric proanthocyanidin complexes: history, structure, and phytopharmaceutical applications. Alternative Med. Rev..

[3] Clouatre D.L., Kandaswami C., Connolly K.M., Coates P.M., Betz J.M., Blackman M.R. (2010). Encyclopedia of Dietary Supplements.

[4] Zhang H., Liu S., Li L. (2016). The impact of grape seed extract treatment on blood pressure changes: a meta-analysis of 16 randomized controlled trials. Medicine (Baltim.).

[5] Nair N., Mahajan S., Chawda R., Kandaswami C., Shanahan T.C., Schwartz S.A. (2002). Grape seed extract activates Th1 cells in vitro. Clin. Diagn. Lab. Immunol..

[6] Konowalchuk J., Speirs J.I. (1976). Virus inactivation by grapes and wines. Appl. Environ. Microbiol..

[7] Lee Y.H. (2017). Green tea catechin-inactivated viral vaccine platform. Front. Microbiol..

[8] Beyer W.E., Palache A.M., Osterhaus A.D. (1998). Comparison of serology and reactogenicity between influenza subunit vaccines and whole virus or split vaccines: a review and meta-analysis of the literature. Clin. Drug Invest..

[9] Sanders B., Koldijk M., Schuitemaker H., Nunnally B., Turula V., Sitrin R. (2014). Vaccine Analysis: Strategies, Principles, and Control.

[10] Geeraedts F., Ter Veer W., Wilschut J., Huckriede A., de Haan A. (2012). Effect of viral membrane fusion activity on antibody induction by influenza H5N1 whole inactivated virus vaccine. Vaccine.

[11] Uittenbogaard J.P., Zomer B., Hoogerhout P., Metz B. (2011). Reactions of beta-propiolactone with nucleobase analogues, nucleosides, and peptides: implications for the inactivation of viruses. J. Biol. Chem..

[12] Sidwell R.W., Bailey K.W., Wong M.H., Barnard D.L., Smee D.F. (2005). In vitro and in vivo influenza virus-inhibitory effects of viramidine. Antivir. Res..

[13] Reed L.J., Muench H. (1938). A simple method of estimation of fifty per cent endpoints. Am. J. Hyg..

[14] Nerome K. (2015). The large-scale production of an artificial influenza virus-like particle vaccine in silkworm pupae. Vaccine.

[15] Fattom A. (2004). Safety and immunogenicity of a booster dose of Staphylococcus aureus types 5 and 8 capsular polysaccharide conjugate vaccine (StaphVAX) in hemodialysis patients. Vaccine.

[16] Barnard D.L., Wong M.-H., Bailey K., Day C.W., Sidwell R.W., Hickok S.S., Hall T.J. (2007). Effect of oral gavage treatment with ZnAL42 and other metallo-ion formulations on influenza A H5N1 and H1N1 virus infections in mice. Antivir. Chem. Chemother..

[17] Takada A., Kuboki N., Okazaki K., Ninomiya A., Tanaka H., OzHaki S., Itamura H., Nishimura M., Enami M., Tashiro K.F., Shortridge Kida H. (1999). Avirulent Avian influenza virus as a vaccine strain against a potential human pandemic. J. Virol..

[18] Ge H., Liu G., Xiang Y.F., Wang Y., Guo C.W., Chen N.H., Zhang Y.J., Wang Y.F., Kitazato K., Xu J. (2014). The mechanism of poly-galloyl-glucoses preventing Influenza A virus entry into host cells. PLoS One.

[19] Ainai A., Suzuki T., Tamura S.I., Hasegawa H. (2017). Intranasal administration of whole inactivated influenza virus vaccine as a promising influenza vaccine candidate. Viral Immunol..

