# Supplementary material for: Examining the interactions of Galahad™ compound with viruses to develop a novel inactivated influenza A virus vaccine
Source: Heliyon. 2022 Jul 6;8(7):e09887. doi: 10.1016/j.heliyon.2022.e09887 (PMC9258431; doi:10.1016/j.heliyon.2022.e09887)
Supplement: Appendix A. Supplemental material Tables 4 [file mmc1.doc]

**Supplemental Material** for

Examining the interactions of Galahad^TM^ compound with viruses to develop a novel inactivated influenza A virus vaccine

Dale L. Barnard a, David M. Belnap b, Parastoo Azadi c, Christian Heiss c, D. Scott Snyder d, Susan C. Bock e, Thomas W. Konowalchuk f, *

a *ADVS Department, Institute for Antiviral Research, Utah State University, Logan, Utah 84041, United States*b *School of Biological Sciences and Department of Biochemistry, University of Utah, Salt Lake City, Utah 84112, United States*

c *Complex Carbohydrate Research Center, 315 Riverbend Road Athens 30602-4712, Georgia*d *Snyder SutroVax Inc., Foster City, CA, United States*e *Biomedical Engineering Department, University of Utah, Salt Lake City, Utah 84112, United States*f *Galaxy Force Technologies, LLC 1070 NE 7th Drive, Newport, OR 97365-2518, United States*

**Corresponding Author*

**Appendix A. Supplemental material**

## The following are the supplementary data to this article:

I. Figure S1. Structure of the catechin moiety of Galahad^TM^

II. Figure S2. Structural changes of adenovirus virions and influenza virus-like particles after Galahad^TM^ treatment, viewed by negative-stain electron microscopy

III. Figure S3. Representative images of lung pathology

IV. Methods for characterizing Galahad^TM^

V. Method used to make influenza virus-like-particles (VLPs)

VI. Table S1. Molecular weight determination of Galahad^TM^

VII. Table S2. Mol% of sugar in the carbohydrate component

VIII. Table S3. Glycosyl composition analysis

IX. Table S4. NMR chemical shift assignments of arabinan

X. Table S5. Effects of undiluted Galahad^TM^ on infectivity of viruses

XI. Table S6. Effects of Galahad^TM^ diluted 1/10 on infectivity of viruses

XII. Table S7. Effects of Galahad^TM^ diluted 1/100 on infectivity of viruses

XIII. Supplemental literature cited

I. Figure S1. Structure of the catechin moiety of Galahad^TM^


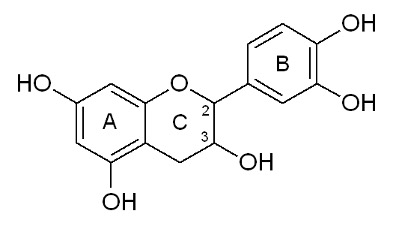


II. Figure S2A. Structural changes of adenovirus virions after Galahad^TM^ treatment (80 µg/mL) viewed by negative-stain electron microscopy at low magnification.


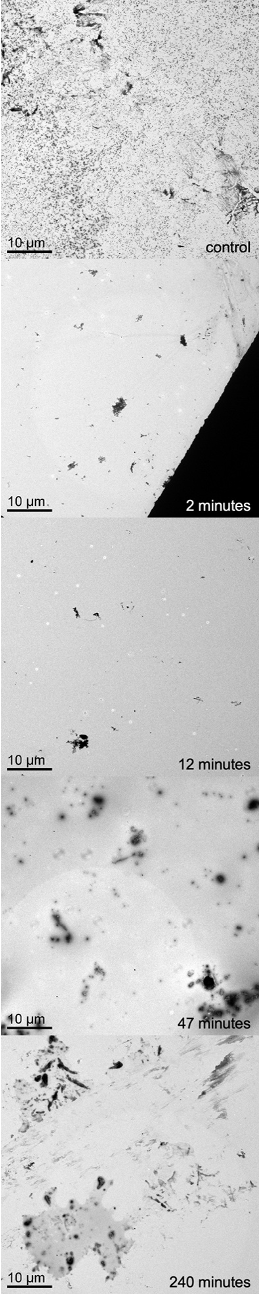


(Top to bottom, respectively) Untreated virus (control), 2-minute Galahad^TM^ treatment, 12-minute Galahad^TM^ treatment, 47-minute Galahad^TM^ treatment, 240-minute Galahad^TM^ treatment.

Figure S2B. Effects of Galahad^TM^ treatment (80 µg/mL) on adenovirus virions after Galahad^TM^ treatment (80 µg/mL) viewed by negative-stain electron microscopy at medium magnification.


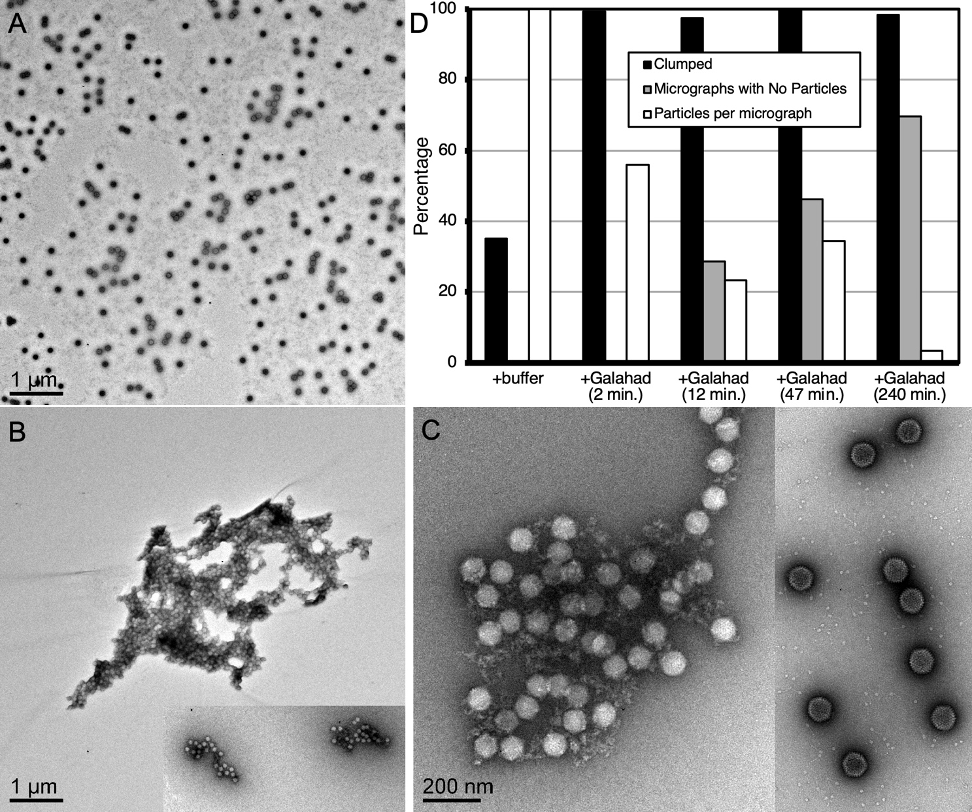


A. Virus particles treated with buffer (1:1), shown at a medium magnification.

B. Galahad^TM^ treated adenovirus after two minutes incubation. Large (panel) and small (inset lower right) clumps were observed, shown at a medium magnification.

C. Higher magnification views of Galahad^TM^-treated virus after a 47-minute incubation time (left) and buffer-treated virus (right).

D. Percentage of clumped particles observed (black bars), micrographs without particles (gray), and particles per micrograph compared to those in the buffer-treated experiment (white bars).

Views of particles similar to those seen in micrographs shown in panels A and B were used to count the particles and to calculate the percentages shown in panel D.

Figure S2C. Structural changes of influenza virus-like particles after Galahad^TM^ treatment (200 µg/mL) viewed by negative-stain electron microscopy at medium and low magnification.


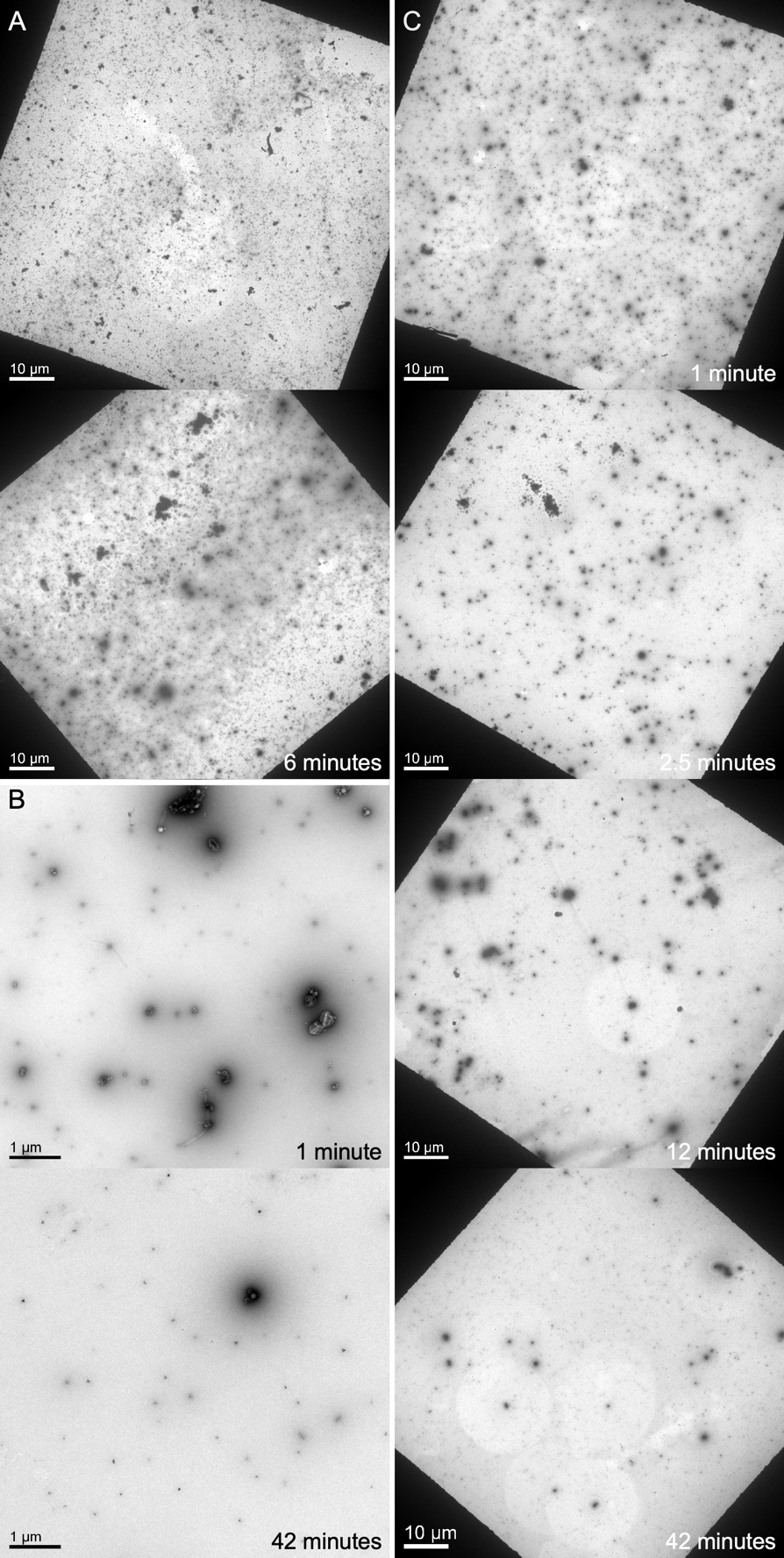


A. Two control experiments where influenza VLP specimens were prepared without treatment (top) or by mixing with buffer (bottom). Specimens were viewed at low magnification.

B,C. Treatment of VLPs with Galahad at a concentration of 200 µg/ml. Incubation time is indicated on each micrograph. Specimens viewed at medium (B) or low (C) magnification.

III. Figure S3. Representative images of lung pathology

A. Mild B. Moderate


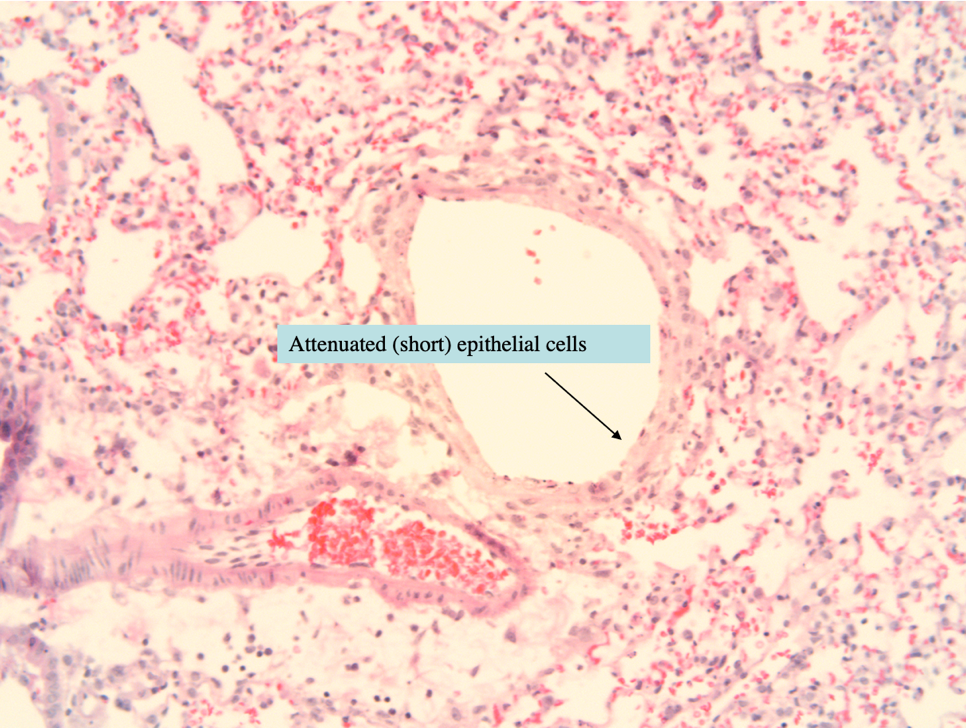

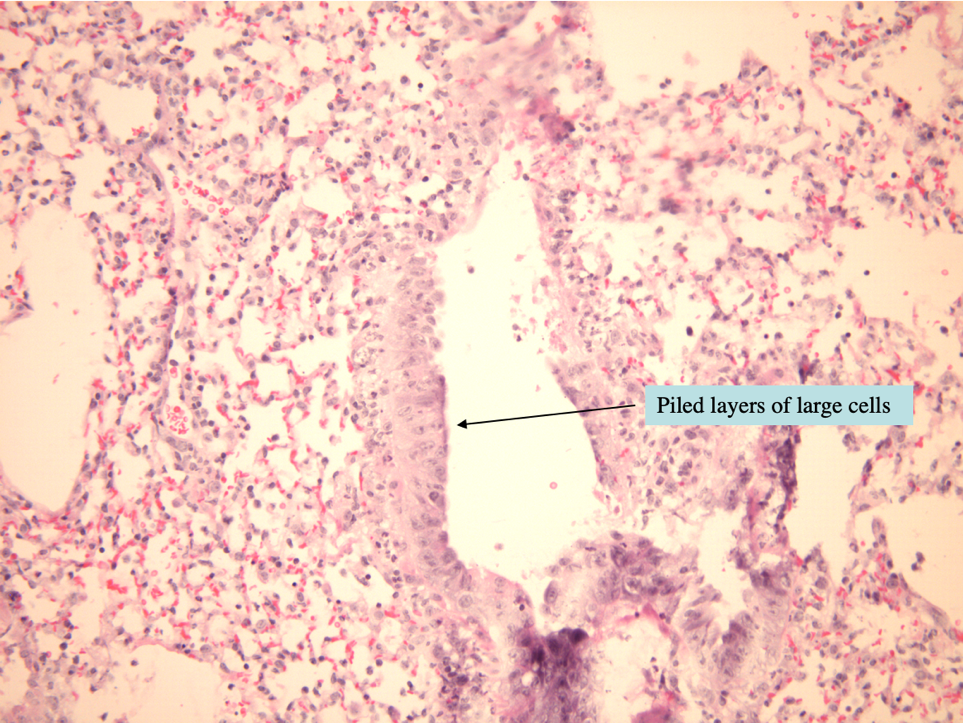


C. Severe D. Normal lung section


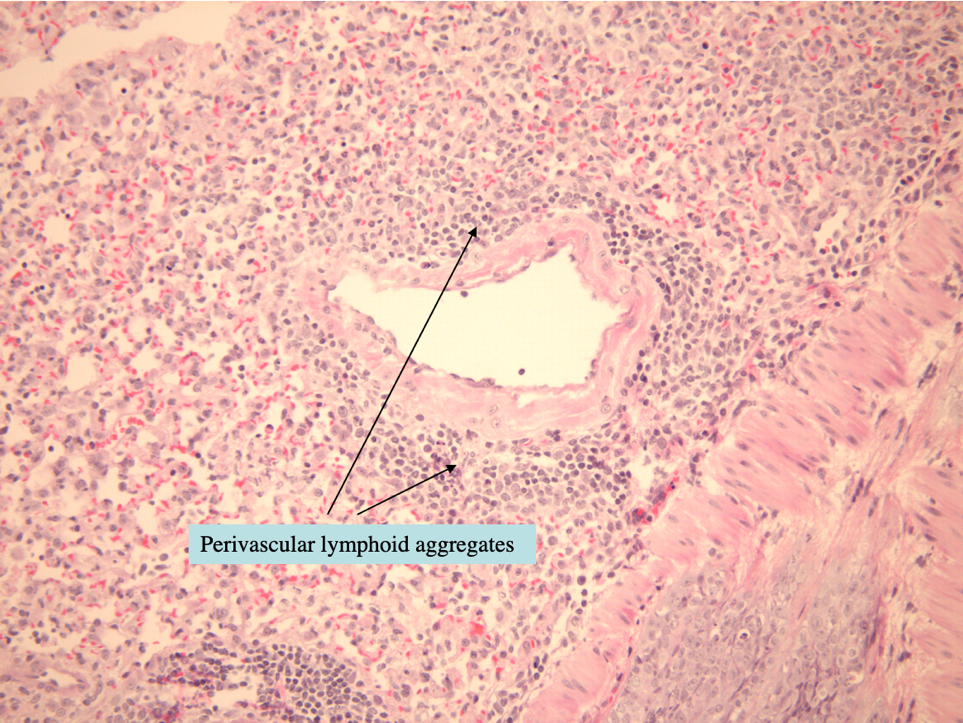

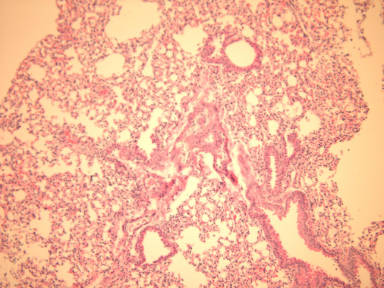


Magnification = 40X

IV**.** Methods for characterizing Galahad^TM^

1.1 Extraction and preparation of Galahad^TM^

Various concentrations of Galahad^TM^ in distilled water were prepared from a 5% grape

seed extract (GSE). For use in cell culture experiments and in vivo vaccine experiments, 18mg/mL Galahad^TM^ was filtered through a micropore filter (0.2-micron, Millipore Sigma, St. Louis, MO, USA) to provide a material free of bacterial contaminants. The production of Galahad^TM^ has been standardized over several hundred batches prepared on the lab bench. Variation of raw material is removed by the DOW Amberlyst 22 Resin column step. The density of Galahad^TM^ is adjusted for mg/mL by photospectrometry testing and potency is tested by viral inactivation assays. Currently this process is being migrated to commercial scale.

Galahad^TM^ was prepared on the lab bench as follows: 75 gms of GSE were dissolved in 1000 mL distilled water using a two-liter beaker on a stirring plate with a two-inch stirring rod at an initial rate of 700 RPM. Slowly the GSE was added so that no lumps appeared. During the adding step, the RPM was increased so that a vortex to the bottom of the beaker was maintained – up to 1000 RPM. Once dissolved, the GSE was divided into two square 500 mL bottles and shaken vigorously fifty times. The contents (500 mL) of each of the two shaking bottle was poured onto one filter paper (Whatman cat No 1213-185 18.5 cm circle). One batch of Galahad^TM^ (400mL of 40mg/mL) used a total volume of dissolved GSE of two liters, thus there was a total of two filter papers used per batch.

After the dissolved GSE passed through the filter paper by gravity, it was divided in two and one half layered on top of a column. The columns are 3.25 inch diameter, 24 inches long, with a number four stopcock. A plug of glass wool was placed in the bottom and the column was filled with a slurry of DOW Amberlyst 22 Resin (was XUS-43568) to about seven inches from the top (800 mL of Resin in each column for 1000 mL of dissolved GSE). A total of 1000 mL was added to each column, starting with 500 mL onto each column. The drip rate was set so that individual drops dripped at the rate of one drop every three seconds (it took four hours for the 1000 mL to pass trough the column). When the exiting drops had a red color, the collection was started. 100 mL of distilled water was added to each column when the fluid finally reached the top of the slurry. The collection stopped when the drops lost the red color or the column stopped dripping. There was about 1000-1200 mL collected for each column.

The collections from the columns were pooled and compressed until the volume was approximately 300 mL using a dialysis unit with 18 Pali Minimate TFF Capsules (500kDa pore size) in parallel. The pump flow rate was set to approximately 80-100 mL/minute. The back pressure for each Capsule was set to 10 PSI so there was a good drip rate from the filtrate output. After compression, the product was washed with 1000 mL distilled water using a continuous drip addition technique.

The final product from each batch was tested for the concentration of purified Galahad^TM^ using a photospectrometer (Genesys 10S UV-Vis) at 439NM (linear equation y = 0.5900995 x -8.583618E-03) and distilled water was added to adjust each batch to the same concentration. Each batch was evaluated for composition to eliminate any batch to batch variation. In addition, each batch was tested in a virus neutralization assay in cell culture to determine if the virus neutralization profiles were equal from batch to batch (less than 1 log_10_ variation in virus neutralization titers).

2.2 Compound characterization

*Glycosyl composition analysis*

Glycosyl composition analysis was performed by combined gas chromatography/mass

spectrometry (GC/MS) of the per-O-trimethylsilyl (TMS) derivatives of the monosaccharide methyl glycosides produced from the sample by acidic methanolysis. Methyl glycosides were first prepared from a dry sample of Galahad^TM^ by methanolysis in 1 M HCl in methanol at 80^o^C (18-22 hours), followed by re-N-acetylation with pyridine and acetic anhydride in methanol (for detection of amino sugars). The samples were then per-O-trimethylsilylated by treatment with Tri-Sil (Pierce) at 80^o^C (0.5 hours). These procedures were carried out as previously described [1,2]. GC/MS analysis of the TMS methyl glycosides was performed on an HP 5890 GC interfaced to a 5970 MSD, using an All-Tech EC-1 fused silica capillary column (30 m x 0.25 mm ID).

*Glycosyl linkage analysis*

For glycosyl linkage analysis, the dialyzed sample was methylated by a modification of the method of Hakomori as described by York et al [2]; depolymerized, reduced, and acetylated; and the resultant partially methylated alditol acetates (PMAAs) analyzed by gas chromatography-mass spectrometry (GC-MS) as described by York et al [2]. Briefly, An aliquot was taken from the sample after lyophilizing and dissolved in DMSO, 0.4 mL potassium dimsylate (2.0 M) was added. After seven hours at room temperature on stirrer, the reaction mixture was cooled to 0^o^C, excess methyl iodide (0.7 mL) was added, and the tube sealed. Incubation was then continued for 24 hours at room temperature. Following sample workup, the permethylated material was hydrolyzed using 2 M trifluoroacetic acid (three hours in sealed tube at 100^o^C), reduced with NaBD_4_ and acetylated using acetic anhydride/pyridine. The resulting PMAAs were analyzed on a Hewlett Packard 5890 GC interfaced to a 5970 MSD (mass selective detector, electron impact ionization mode); separation was performed on a 30 m Supelco 2330 bonded phase fused silica capillary column.

*Dynamic light scattering*

Dextran standards (40, 167, 511, and 1400 kDa) and ammonium sulfate precipitated

and dialyzed Galahad^TM^ were prepared as two mg/mL solutions in water. The raw Galahad^TM^ solution was diluted 5-fold with water. The samples were centrifuged for 15 minutes before analysis to remove particulates.

Measurements were carried out on a Protein Solutions DynaPro 99 dynamic light scattering machine at 25^o^C, using the Dynamics software, version 6.03. Laser power was set to 50% (standards) and 30% (samples), respectively. Sampling interval was every 5 seconds and a minimum of 20 measurements was collected and averaged.

*Amino acid composition*

Amino acid composition analysis was performed by combined gas chromatography/mass spectrometry (GC/MS) of the heptafluorobutyrate (HFB) derivatives of the amino acid isoamyl esters produced from the sample by acidic transesterification as per the method of Pons et al. [3]. Samples were first hydrolyzed in 6 M HCl overnight at 110^o^C. one nmol of norleucine internal standard was then added and samples were dried under a gentle stream of nitrogen. Samples were then resuspended in 0.5 M methanolic HCl and incubated at 80^o^C overnight. Transesterification was then performed in 1.5 M isoamyl HCl at 100^o^C overnight followed by evaporation under nitrogen and acylation with heptafluorobutyric acid anhydride (50 μl in 200 μl acetonitrile). GC/MS analysis of the amino acid derivatives was performed on an HP 5890 GC interfaced to a 5970 MSD, using a CP Sil5 capillary column (30 m 0.25 mm ID) and a temperature gradient of 90 to 260^o^C at 5^o^C/min.

*Phloroglucinolysis*

Two different methods of phloroglucinolysis were used. For breakdown of the components of a standard compound, Carlo Rossi Burgundy, an incubation in 1 M HCl in methanol at 80^o^C for 2 hours in the presence of 50mg phloroglucinol was used. All hydrolyses of Galahad^TM^ Red were made using 50 mg/ml phloroglucinol in 1 M HCl in methanol 80^o^C with overnight incubation. Reactions were analyzed by TLC on silica gel TLC plates using chloroform methanol 9:1 and in some cases chloroform methanol 0.25% KCl 5:4:1 as a developing solvent. Visualization was accomplished either by the use of a UV lamp and by staining with ceric ammonium nitrate/ammonium molybdate in ethanol sulfuric acid.

*NMR Spectroscopy*

The sample was dialyzed, freeze-dried, and deuterium-exchanged by lyophilization from

D_2_O and dissolved in 0.7 mL D_2_O. 1-D Proton and 2-D gCOSY, TOCSY, NOESY, and

gHSQC spectra were acquired on a Varian Inova-500 MHz spectrometer at 298^o^K (25^o^C) using standard Varian pulse sequences. The TOCSY and NOESY mixing times were set to 80 and 300 ms, respectively. Proton chemical shifts were measured relative to internal acetone (δH=2.225 ppm, δC=31.07 ppm).

V. Methods used to make influenza virus-like-particles (VLPs)

Preparation of influenza VLPs (virus-like-particles) in *T. ni* pupae Living Biofactory System

Virus-like particles (VLP) were used to study the effects of Galahad^TM^ exposure on influenza virus surface proteins. Briefly, *Trichoplusia .ni* pupae were injected on ~d2 post spinning with 8 μL containing ~1000 pfu and ~100 pfu, respectively, of *Autographa californica multiple nucleopolyhedrovirus* baculovirus stocks for the expression of recombinant H5N1 influenza A/Vietnam/1203/2004 hemagglutinin (HA) and M1 matrix protein 1. After 80 hours at 27°C, the pupae were frozen at -20°C. The VLP purification protocol was modified from isolation of flu HA VLPs from *Bombyx mori* pupae by Nerome [4]. A pupal extract was prepared in ice-cold phosphate buffered saline (PBS) pH 7.4 containing 500 mM NaCl, 2 mM PMSF, 10% formalin, and saturating PTU (phenylthiourea, a melaninization inhibitor). A VLP-enriched fraction obtained by differential centrifugation of the crude extract at 2000 x g for 10 minutes was pelleted from the low-speed supernatant at 14,000 x g for 25 minutes. The VLP pellet was resuspended in PBS 7.4/500 mM NaCl by 12 passes through a 1.5 inch 22-gauge needle. Resuspended VLPs were layered on top of sucrose step gradients prepared from 20%, 40% and 60% sucrose in PBS 7.4/500 mM NaCl, and centrifuged at 97,000 x g for 14 minutes (Beckman Airfuge, A95 rotor). VLPs were recovered from the 40-60% sucrose interface.

VI. Table S1. Molecular weight determination of Galahad^TM^ using dynamic light scattering

| Sample | r (nm) | % polydispersity | MW (kDa) | % mass |
| --- | --- | --- | --- | --- |
| Galahad^TM^ | 6.1 | 13.0 | 68 | >95 |
|  | 25.6 | 14.0 | 1,600 | <5 |
| AS ppt | 5.4 | 24.6 | 54 | >95 |
|  | 28.5 | 21.6 | 2,200 | <5 |

VII. Table S2. Mol% of sugar in the carbohydrate component.

| Sugar | Low % | High % | Mean ± 3SD |
| --- | --- | --- | --- |
| Arabinose (Acs ra) | 33.2 | 74.2 | 53.7 ± 20.5 |
| Rhamnose (Rha) | 0 | 9.3 | 4.2 ± 5.1 |
| Xylose (Xyl) | 0 | 4.4 | 1.7 ± 2.7 |
| Glucuronic Acid (GlcUA) | 0 | 7.3 | 1.7 ± 5.6 |
| Galacturonic acid (GalUA) | 3.4 | 35.8 | 19.6 ± 16.2 |
| Mannose (Man) | 0 | 6.0 | 2.9 ± 3.1 |
| Galactose (Gal) | 1.9 | 19.5 | 10.7 ± 8.8 |
| Glucose (Glc) | 0 | 12.5 | 5.4 ± 7.1 |

VIII. Table S3. Glycosyl composition analysis

| Glycosyl Residue | % |
| --- | --- |
| Terminally linked Rhamnopyranosyl residue (t-Rha) | 1.7 |
| Terminally linked Arabinofuranosyl residue (t-Araf) | 11.6 |
| Terminally linked Fucopyranosyl residue (t-Fuc) | 0.1 |
| Terminally linked Arabinopyranosyl residue (t-Ara) | 1.0 |
| Terminally linked Xylopyranosyl residue (t-Xyl) | 0.8 |
| 2 linked Rhamnopyranosyl residue (2-Rha) | 3.5 |
| Terminally linked Manopyranosyl residue & 4 linked Rhamnopyranosyl residue (t-Man + 4-Rha) | 2.6 |
| Terminally linked Glucopyranosyl residue & Terminally linked Glucuronic Acid residue (t-Glc + t-GlcA) | 3.3 |
| 3 linked Arabinofuranosyl residue (3-Araf) | 3.9 |
| Terminally linked Galactopyranosyl residue & Terminally linked Galacturonic Acid residue (t-Gal + t GalA) | 8.7 |
| 4 linked Arabinopyranosyl residue or 5 linked Arabinofuranosyl residue (4-Arap or 5-Araf) | 14.6 |
| 4 linked Xylopyranosyl residue (4-Xyl) | 1.1 |
| 2,3-Rhamnopyranosyl residue (2,3-Rha) | 0.1 |
| 2,4-Rhamnopyranosyl residue (2,4-Rha) | 2.4 |
| 2 linked Manopyranosyl residue (2-Man) | 3.0 |
| 2 linked Glucopyranosyl residue & 2 linked Glucuronic Acid residue (2-Glc + 2-GlcA) | 0.4 |
| 3 linked Galactopyranosyl residue (3-Gal) | 2.9 |
| 4 linked Manopyranosyl residue (4-Man) | 1.1 |
| 3,4 linked Arabinopyranosyl or 3,5 linked Arabinofuranosyl (3,4-Arap or 3,5-Araf) | 6.4 |
| 6 linked Glucuronic Acid residue & 6 linked Glucopyranosyl residue (6-GlcA + 6-Glc) | 0.5 |
| 4 linked Galacturonic Acid residue & 4 linked Galactopyranosyl residue (4-GalA + 4-Gal) | 7.5 |
| 2,4 linked Arabinopyranosyl or 2,5 linked Arabinofuranosyl (2,4-Arap or 2,5-Araf) | 1.4 |
| 4 linked Glucuronic Acid residue & 4 linked Glucopyranosyl residue (4-GlcA + 4-Glc) | 7.3 |
| 2,3 linked Manopyranosyl residue (2,3-Man) | 0.9 |
| 6 linked Galactopyranosyl residue (6-Gal) | 1.1 |
| 2,3,4 linked Arabinopyranosyl residue (2,3,4-Ara) | 4.0 |
| 3,4 linked Galacturonic Acid residue & 3,4 linked Galactopyranosyl residue (3,4-Gal + 3,4-GalA) | 1.2 |
| 3,4 linked Glucuronic Acid residue & 3,4 linked Glucopyranosyl residue (3,4-Glc + 3,4-GlcA) | 0.4 |
| 2,4 linked Manopyranosyl residue (2,4-Man) | 0.1 |
| 2,4 linked Galacturonic Acid residue & 2,4 linked Galactopyranosyl residue (2,4-GalA + 2,4-Gal) | 0.9 |
| 4,6 linked Manopyranosyl residue (4,6-Man) | 0.1 |
| 4,6 linked Glucopyranosyl residue & 4,6 linked Glucuronic Acid residue (4,6-Glc + 4,6-GlcA) | 0.5 |
| 4,6 Galacturonic acid residue & 4,6-Galactopyranosyl residue (4,6-GalA + 4,6-Gal) | 0.5 |
| 3,6 linked Galactopyranosyl residue (3,6-Gal) | 4.1 |

^1^The glycosyl composition analysis reported above does NOT include the absolute configuration (D or L) of monosaccharides.

IX. Table S4. NMR Chemical shift assignments of arabinan

| Residue |  | Chemical Shift (ppm) |  | | | | | |
| --- | --- | --- | --- | --- | --- | --- | --- | --- |
|  |  | 1 | | 2 | 3 | 4 | 5 | 5’ |
| t-α-Ara*f* | *^1^H* | 5.21 | | 4.17 | 3.99 | 4.08 | 3.85 | 3.74 |
|  | *^13^C* | 108.0 | | 82.0 | 77.5 | 84.8 | 61.8 |  |
| t-α-Ara*f* | *^1^H* | 5.18 | | 4.17 | 3.98 | n.d. | n.d. | n.d. |
|  | *^13^C* | 107.8 | | 82.0 | 77.5 | n.d. | n.d. |  |
| 5-α-Ara*f* | *^1^H* | 5.07 | | 4.24 | 3.92 | 4.06 | 3.97 | 3.79 |
|  | *^13^C* | 108.2 | | 82.0 | 77.5 | 82.0 | n.d. |  |
| 3,5-α-Ara*f* | *^1^H* | 5.10 | | 4.16 | 4.07 | 4.24 | 3.97 | 3.83 |
|  | *^13^C* | 108.2 | | 82.0 | 82.7 | 82.9 | 67.2 |  |
| 2,5-α-Ara*f* | *^1^H* | 5.27 | | 4.24 | 4.06 | n.d. | 3.97 | 3.83 |
|  | *^13^C* | 107.3 | | 86.2 | n.d. | n.d. | 67.2 |  |
| 2,3,5-α-Ara*f* | *^1^H* | 5.14 | | 4.32 | n.d. | n.d. | 3.97 | 3.83 |
|  | *^13^C* | 108.2 | | 86.2 | n.d. | n.d. | 67.2 |  |
| β-Gal*p* | *^1^H* | 4.52 | | 3.39 | 3.56 | 3.69 | 3.76 | n.d. |

X. Table S5. Effects of **undiluted** Galahad^TM^ on infectivity of viruses

| Virus Family | Virus | | Strain | | Virus Titer with no  Treatment | | Virus Titer with  Galahad^TM^ Treatment | | Log_10_ Reduction in Virus Titer | |
| --- | --- | --- | --- | --- | --- | --- | --- | --- | --- | --- |
| **Non-Enveloped viruses**^a^ |  | |  | |  | |  | |  | |
| Adenoviridae | Adenovirus 1 | | Chicago | | 3.50 | | 0^b^ | | 3.50 | |
| Picornaviridae | Polio virus 1 | | Chat | | 4.50 | | 0^b^ | | 4.50 | |
| Picornaviridae | Rhinovirus 2 | | HGP | | 5.50 | | 0^b^ | | 5.50 | |
| Reoviridae | Human rotavirus | | Wa | | 2.25 | | 0^b^ | | 2.25 | |
| **Enveloped viruses^a^** |  | |  | | | | | | | |
| Arenaviridae | Tacaribe | | TV | | 4.50 | | 0^b^ | | 4.50 | |
| Bunyaviridae | Rift Valley Fever (vaccine) | | MP-12 | | 6.25 | | 0^b^ | | 6.25 | |
| Coronaviridae | SARS-CoV-1 | | Urbani | | 4.25 | | 0^a^ | | 4.25 | |
| Coronaviridae | | SARS-CoV-2 | | USA-WA1/2020 | | 5.3 | | 2.5 | | 2.80 |
| Herpesviridae | Herpes Simplex 1 | | McIntyre | | 4.75 | | 0^b^ | | 4.75 | |
| Orthomyxoviridae | Influenza A H3N2 | | A/California/7/04 | | 3.50 | | 0^b^ | | 3.50 | |
| Orthomyxoviridae | Influenza A H1N1 | | A/New Caledonia/20/99 | | 4.25 | | 0^b^ | | 4.25 | |
| Orthomyxoviridae | Influenza A H5N1 | | Vietnam/1203/4 X Ann Arbor/6/60 | | 2.75 | | 0^b^ | | 2.75 | |
| Orthomyxoviridae | Influenza A H5N1 | | Vietnam/1203/4 X Ann Arbor/6/60 (oseltamivir resistant) | | 3.75 | | 0^b^ | | 3.75 | |
| Orthomyxoviridae | Influenza A H5N1 | | Duck/MN/1525/81 | | 3.50 | | 0^b^ | | 3.50 | |
| Orthomyxoviridae | Influenza B | | B/Shanghai/361/02 | | 2.75 | | 0^b^ | | 2.75 | |
| Paramyxoviridae | Measles | | MO-6 | | 5.50 | | 0^b^ | | 5.50 | |
| Paramyxoviridae | Parainfluenza virus 3 | | 14709 | | 3.75 | | 0^b^ | | 3.75 | |
| Paramyxoviridae | Respiratory syncytial virus | | A1 | | 4.75 | | 0^b^ | | 4.75 | |
| Poxviridae | Vaccinia virus | | Copenhagen | | 4.50 | | 0^b^ | | 4.50 | |
| Rhabdoviridae | Vesicular stomatitis virus | | Indiana | | 4.75 | | 0^b^ | | 4.75 | |
| Flaviviridae | Dengue Fever virus | | New Guinea | | 4.50 | | 3.50 | | 1.00 | |
| Flaviviridae | Yellow Fever virus | | 17D | | 4.50 | | 1.50 | | 3.00 | |
| Flaviviridae | West Nile virus | | New York | | 6.70 | | 0^b^ | | 6.70 | |

^a^Virus was exposed to Galahad^TM^ for 10 minutes at room temperature.

^b^ Below the limits of detection of the assay.

**P<0.001

XI. Table S6. Effects of Galahad^TM^ **diluted 1/10** on infectivity of viruses

| Virus Family | Virus | | Strain | | Virus Titer with no  Treatment | Virus Titer with  Galahad^TM^ Treatment | Log_10_ Reduction in Virus Titer | | |
| --- | --- | --- | --- | --- | --- | --- | --- | --- | --- |
| **Non-Enveloped viruses**^a^ | | |  | | | | |  |  |
| Adenoviridae | Adenovirus 1 | | Chicago | | 3.50 | 0^b^ | 3.50 | | |
| Picornaviridae | Polio virus 1 | | Chat | | 4.50 | 1.50 | 3.00 | | |
| Picornaviridae | Rhinovirus 2 | | HGP | | 5.50 | 4.50 | 1.00 | | |
| Reoviridae | Human rotavirus | | Wa | | 2.25 | 1.50 | 0.75 | | |
| **Enveloped viruses** | | |  | | | | |  |  |
| Arenaviridae | Tacaribe | | TV | | 4.50 | 4.75 | 0.00 | | |
| Bunyaviridae | Rift Valley Fever (vaccine) | | MP-12 | | 6.25 | 6.25 | 0.00 | | |
| Coronaviridae | SARS-CoV-1 | | Urbani | | 4.25 | 2.25 | 2.00 | | |
| Coronaviridae | | SARS-CoV-2 | | USA-WA1/2020 | 5.3 | 5.3 | 0.00 | | |
| Herpesviridae | Herpes Simplex 1 | | McIntyre | | 4.75 | 3.75 | 1.00 | | |
| Orthomyxoviridae | Influenza A H3N2 | | A/California/7/04 | | 3.50 | 0^b^ | 3.50 | | |
| Orthomyxoviridae | Influenza A H1N1 | | A/New Caledonia/20/99 | | 4.25 | 0^b^ | 4.25 | | |
| Orthomyxoviridae | Influenza A H5N1 | | Vietnam/1203/4 X Ann Arbor/6/60 | | 2.75 | 0^b^ | 2.75 | | |
| Orthomyxoviridae | Influenza A H5N1 | | Vietnam/1203/4 X Ann Arbor/6/60 (oseltamivir resistant) | | 3.75 | 0^b^ | 3.75 | | |
| Orthomyxoviridae | Influenza A H5N1 | | Duck/MN/1525/81 | | 3.50 | 0^b^ | 3.50 | | |
| Orthomyxoviridae | Influenza B | | B/Shanghai/361/02 | | 2.75 | 0^b^ | 2.75 | | |
| Paramyxoviridae | Measles | | MO-6 | | 5.50 | 3.50 | 2.00 | | |
| Paramyxoviridae | Parainfluenza virus 3 | | 14709 | | 3.75 | 1.25 | 2.50 | | |
| Paramyxoviridae | Respiratory syncytial virus | | A1 | | 4.75 | 3.75 | 1.00 | | |
| Poxviridae | Vaccinia virus | | Copenhagen | | 4.50 | 0^b^ | 4.50 | | |
| Rhabdoviridae | Vesicular stomatitis virus | | Indiana | | 4.75 | 0^b^ | 4.75 | | |

^a^Virus was exposed to Galahad^TM^ for 10 minutes at room temperature.

^b^ Below the limits of detection of the assay.

XII. Table S7. Effects of Galahad^TM^ **diluted 1/100** on infectivity of viruses

| Virus Family | Virus | Strain | Virus Titer with no  Treatment | Virus Titer with  Galahad^TM^ Treatment | Log_10_ Reduction in Virus Titer | |
| --- | --- | --- | --- | --- | --- | --- |
| **Non-Enveloped viruses**^a^ | |  | | |  |  |
| Adenoviridae | Adenovirus 1 | Chicago | 3.50 | 0^b^ | 3.50 | |
| Picornaviridae | Polio virus 1 | Chat | 4.50 | 1.50 | 3.00 | |
| Picornaviridae | Rhinovirus 2 | HGP | 5.50 | 5.75 | 0.00 | |
| Reoviridae | Human rotavirus | Wa | 2.25 | 1.50 | 0.75 | |
| **Enveloped viruses**^a^ | |  | | | | |
| Arenaviridae | Tacaribe | TV | 4.50 | 5.25 | 0.00 | |
| Bunyaviridae | Rift Valley Fever (vaccine) | MP-12 | 6.25 | 7.25 | 0.00 | |
| Coronaviridae | SARS-CoV-1 | Urbani | 4.25 | 3.50 | 0.75 | |
| Coronaviridae | SARS-CoV-2 | USA-WA1/2020 | 5.30 | 5.30 | 0.00 | |
| Herpesviridae | Herpes Simplex  1 | McIntyre | 4.75 | 4.50 | 0.25 | |
| Orthomyxoviridae | Influenza A H3N2 | A/California/7/04 | 3.50 | 1.50 | 2.00 | |
| Orthomyxoviridae | Influenza A H1N1 | A/New Caledonia/20/99 | 4.25 | 1.25 | 3.00 | |
| Orthomyxoviridae | Influenza A H5N1 | Vietnam/1203/4 X Ann Arbor/6/60 | 2.75 | 0.75 | 2.00 | |
| Orthomyxoviridae | Influenza A H5N1 | Vietnam/1203/4 X Ann Arbor/6/0 (oseltamivir resistant) | 3.75 | 3.25 | 0.50 | |
| Orthomyxoviridae | Influenza A H5N1 | Duck/MN/1525/81 | 3.50 | 1.75 | 1.75 | |
| Orthomyxoviridae | Influenza B | B/Shanghai/361/02 | 2.75 | 0^b^ | 2.75 | |
| Paramyxoviridae | Measles | MO-6 | 5.50 | 5.75 | 0.00 | |
| Paramyxoviridae | Parainfluenza virus 3 | 14709 | 3.75 | 2.75 | 1.00 | |
| Paramyxoviridae | Respiratory syncytial virus | A1 | 4.75 | 4.25 | 0.50 | |
| Poxviridae | Vaccinia virus | Copenhagen | 4.50 | 2.25 | 2.25 | |
| Rhabdoviridae | Vesicular stomatitis virus | Indiana | 4.75 | 1.25 | 3 | |
|  |  |  |  |  |  | |

| ^a^Virus was exposed to Galahad^TM^ for 10 minutes at room temperature.^b^ Below the limits of detection of the assay |
| --- |

XIII. Supplemental Literature Cited

[1] Merkle RK and Poppe I. Carbohydrate composition analysis of glycoconjugates by gas-liquid chromatography/mass spectrometry. Methods Enzymol. 1994; 230:1-15. DOI: [10.1016/0076-6879(94)30003-8](#_blank).

[2] York WS, Darvill AG, McNeil M, Stevenson TT, Albersheim P. Isolation and characterization of plant cell walls and cell wall components. Methods Enzymol. 1985; 118:3-40. DOI: <https://doi.org/10.1016/0076-6879(86)18062-1>.

[3] Pons A, et al. Sequential GC/MS analysis of sialic acids, monosaccharides, and amino acids of glycoproteins on a single sample as heptafluorobutyrate derivatives. Biochemistry. 2003; 42: 8342-8353. DOI: [10.1021/bi034250e](#_blank).

[4] Nerome K, et al. The large-scale production of an artificial influenza virus-like particle vaccine in silkworm pupae*.* Vaccine, 2015; 33:117-125. DOI: [10.1016/j.vaccine.2014.11.](#_blank)
